# Emerging ozone generation strategies: mechanistic insights and application-driven developments

**DOI:** 10.1039/d6ra01992g

**Published:** 2026-05-06

**Authors:** Fekri Abdulraqeb Ahmed Ali, Hisham Khalid alsmail, T. Vinod Kumar, J. R. Deepak, Yaqoub Abdu Hakami, S. Padmanabhan, Amine Aymen Assadi, P. K. Kishore Kumar, Farid Fadhillah, P. Saravanan, Mohammod Hafizur Rahman, K. Vijayalakshmi, G. Shoba, P. Tamizhdurai

**Affiliations:** a Chemical Engineering Department, College of Engineering, Imam Mohammad Ibn Saud Islamic University (IMSIU) Riyadh 11432 Saudi Arabia; b Hydrogen Technologies Institute, King Abdulaziz City for Science & Technology (KACST) Saudi Arabia; c Department of Mechanical Engineering, Vels Institute of Science, Technology & Advanced Studies (VISTAS) Chennai India; d School of Mechanical Engineering Sathyabama Institute of Science and Technology Chennai – 119 India; e Department of Mechanical Engineering, Vel Tech Rangarajan Dr Sagunthala R&D Institute of Science and Technology Avadi Chennai-62 India; f Department of Mathematics, Jerusalem College of Engineering Narayanapuram, Pallikaranai Chennai – 600100 India; g Department of Chemistry, St. Joseph's College of Engineering OMR Chennai-600119 India; h Department of Biochemistry, Faculty of Science and Humanities, SRM Institute of Science and Technology Kattankulathur Chennai-603203 Tamilnadu India; i Department of Biotechnology, Dwaraka Doss Goverdhan Doss Vaishnav College (Autonomous)(Affiliated to the University of Madras, Chennai) 833, Gokul Bagh, E.V.R. Periyar Road, Arumbakkam Chennai 600 106 Tamil Nadu India; j Department of Chemistry, Dwaraka Doss Goverdhan Doss Vaishnav College (Autonomous)(Affiliated to the University of Madras, Chennai) 833, Gokul Bagh, E.V.R. Periyar Road, Arumbakkam Chennai 600 106 Tamil Nadu India p.tamizhdurai@dgvaishnavcollege.edu.in

## Abstract

Ozone finds widespread use in water and wastewater treatment, air purification, food preservation, and medical sanitation since it has a very high oxidative potential and it decays easily to produce oxygen without leaving any long-lasting residues. However, in practice, the use of ozone on a large scale and over long periods is often limited due to such practical considerations as high energy use, the possibility of the formation of unwanted oxidation by-products, problems of operational and occupational safety, and restrictions on regulatory standards. This review will provide a critical evaluation of the technologies of ozone generation and its use, with the aspects of environmental protection and process safety taken into consideration. It initially describes the basic physical and chemical properties of ozone, the most commonly used methods of its measurement, monitoring and safe handling. Then, the key methods of ozone generation, *i.e.*, ultraviolet irradiation, dielectric barrier discharge, and electrochemical processes are analyzed and compared systematically in the light of their working principles, system design, energy efficiency, economic viability throughout the system operation, and regarding their safety issues. An assignment of practical application cases is then conducted in order to show performance constraints to realistic operating environments. Lastly, the review presents significant barriers to large-scale deployment, including scale-up, system integration and safety management, and points to future directions of coming up with ozone technologies that are safer, more energy-efficient, and more sustainable.

## Abstract 2

Ozone is used for strong oxidation and clean decomposition, but large-scale use is limited by energy demand, by-products, and safety. This review compares generation methods and highlights efficiency and sustainable challenges.

## Graphical abstract

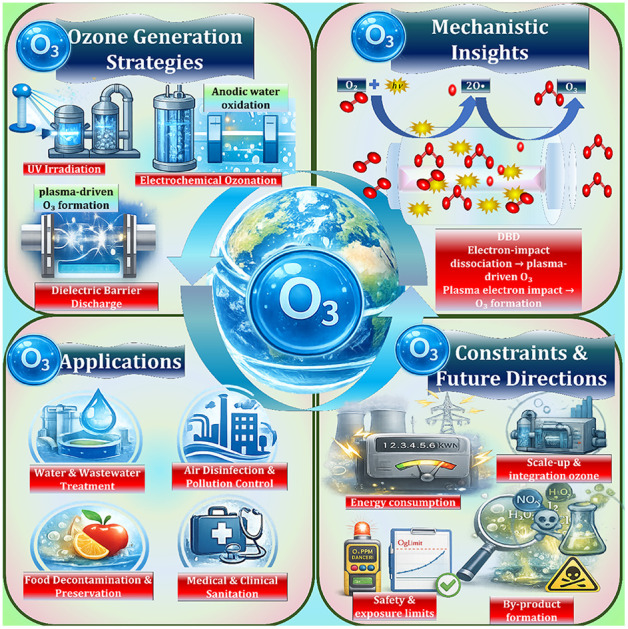

## 1. Introduction

Environmental management technologies increasingly emphasize energy efficiency, operational safety, and minimization of secondary environmental impacts.^1^ More focus is currently being laid on the decrease of energy demand, safe functioning, and minimization of secondary environmental hazards. It is in this respect that advanced oxidation processes (AOPs) have received a lot of attention due to their high ability to break up stubborn and chemically resistant pollutants. Ozone (O_3_) with its high oxidation capacity and speedy reaction mode has been extensively utilized in its application in terms of water and wastewater treatment, air quality management, medical hygiene and industrial disinfection.^2^ Ozonation when regulated can significantly reduce the growth of disinfection by-products that are halogenated as compared to standard methods of chlorine use. Moreover, ozone has also proven to be very effective in the degradation of upcoming micropollutants such as pharmaceutical acids and endocrine disrupters as well as the rapid and efficient inactivation of pathogens.^3^ However, default ozone-based technologies are not sustainable. Their environmental friendliness is also directly related to the level of the efficiency of ozone generation, energy usage in general, the possibility to control oxidation by-products, and the introduction of the overall process safety and risk management practices.^4^

Although the potential of ozone in industrial and environmental use is acknowledged, a significant number of technical and safety-related factors limit the extensive use of the technology. Ozone is volatile and very reactive in nature hence long-term storage and transportation over a long distance is impractical. Thus, it is common to create ozone on the spot instead of centrally.^5^ Dielectric barrier discharge (DBD) generators, which are the most mature and widely-used technologies in industrial ozone generation, are currently used. Nevertheless, they tend to work under high voltage conditions and have relatively low energy efficiency, which makes them more demanding in terms of electrical insulation, equipment lifetime and safety of operation. Moreover, in case of DBD generators working in humid conditions or in the environment with the presence of nitrogen, the evolution of the nitrogen oxides (NO_*x*_) can take place leading to further environmental issues and the complication of the emission control and safety management.^6^ The formation of nitrogen oxides during ozone generation is primarily associated with the activation of nitrogen molecules under high-energy plasma conditions. When air is used as the feed gas in dielectric barrier discharge systems, energetic electrons can dissociate nitrogen and oxygen molecules, initiating reactions that lead to the formation of nitric oxide (NO) and nitrogen dioxide (NO_2_). These compounds may subsequently react with ozone or water vapor to form nitric acid species, which can contribute to equipment corrosion and reduce ozone purity. One of the most effective mitigation strategies is the use of high-purity oxygen rather than ambient air as the feed gas, which significantly suppresses nitrogen participation in plasma reactions. Additional control approaches include improved reactor cooling to limit thermal decomposition reactions, optimization of discharge frequency, and careful control of gas residence time in the plasma region. In aqueous ozonation systems used for drinking water treatment, another potential concern is the formation of bromate (BrO_3_^−^) when bromide ions are present in the source water. Bromate formation typically occurs through oxidation pathways involving bromide ions, hypobromous acid intermediates, and hydroxyl radicals generated during ozone decomposition. Operational strategies to minimize bromate formation include lowering the applied ozone dose, maintaining slightly acidic pH conditions, and introducing ammonia or hydrogen peroxide to alter radical reaction pathways. In addition, pre-treatment methods such as activated carbon adsorption or biological filtration may reduce bromide concentration prior to ozonation, thereby limiting bromate formation and ensuring compliance with drinking water safety regulations.

The development of ozone generation has been at pace with the progress of electrical systems, functional materials and technologies of the plasma, since these areas focus on the failure of the earlier ozone generating techniques.^7^ The modern perspective of the ozone generator first invented by Siemens in the nineteenth century has continuously been oriented on the optimization of electrical power supply, scheme discharges, and dielectric material to enhance the production and reliability of the ozone generator.^8^ Electrochemical ozone generation has become a particularly popular topic in the recent years because of its capability to produce ozone in high concentrations directly in aqueous media. This is a technique that is described by the fact that it forms little NO_*x*_ and the process responds fast to varying loads on the operation, which has obvious benefits over the traditional methods.^9^ Owing to these reasons, electrochemical systems are especially appealing to applications that have small design considerations, high purity of ozone, and very high hygienic standards such as in medical disinfection technologies.^10^ In tandem with this, other methods like the use of UV induced ozone formation and plasma assisted catalytic process have also been considered and offer application specific and flexible solutions to different operating conditions.

Since the ozone technologies are currently used in the field beyond the laboratory-scale experiments, the development of ozone technologies nowadays is determined by the more extended range of engineering considerations instead of the ozone yield. Real-world usage requires the system design to be well-coordinated, safety mechanisms to be strong, and meeting regulatory standards.^11^ Ozone is now being utilized in various industries including tertiary waste water treatment, drinking water sanitation, air quality control and food-processing industries.^12^ The fundamentally differing applications in these applications are their ozone dosage requirements, contact mode and risk management strategies. Consequently, the key issue of real-world ozone systems is the possibility to ensure efficient contaminant removal and at the same time reduce the consumption of energy, take care of operational and occupational safety and minimize the production of unwanted secondary products.^13^

**Fig. 1:**
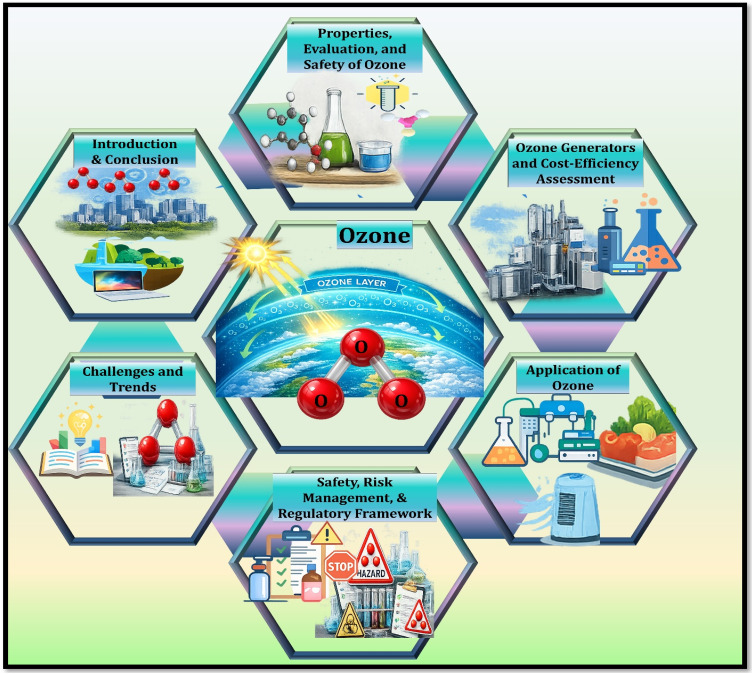
Conceptual structure and content organization of the review.^14^ Adapted from ref. 14 with permission. Copyright (2026) by Elsevier. License number: 6250611493778.

It is against this background that the present review provides a comprehensive and structured analysis of ozone generation technologies and their practical applications. While a number of earlier review studies have discussed ozone generation methods or specific application domains individually, many of them primarily focus on either the fundamental mechanisms of ozone formation or particular technological approaches without providing an integrated assessment of their engineering performance and practical deployment. In contrast, the present review aims to bridge this gap by systematically examining the physicochemical properties of ozone, the operational principles of major ozone generation technologies, and their performance characteristics in real environmental and industrial systems. In particular, this review critically compares the three major ozone generation approaches ultraviolet photochemical systems, dielectric barrier discharge plasma systems, and electrochemical ozone generation by considering not only their reaction mechanisms but also their energy efficiency, operational stability, safety considerations, and economic feasibility. Such a comparative evaluation is essential because the suitability of each technology depends strongly on application requirements, including ozone concentration demand, system scale, energy consumption, and operational reliability. Fig. 1 illustrates the conceptual framework and structural organization of this review, showing how the different components of ozone technology including physicochemical characteristics, generation mechanisms, system performance evaluation, safety management, and application domains—are interconnected. This framework highlights the multidisciplinary nature of ozone technology development, where advances in plasma engineering, electrochemistry, materials science, and environmental engineering collectively influence system performance and applicability. Furthermore, this review places particular emphasis on linking fundamental ozone generation mechanisms with real-world engineering considerations, including process safety, operational constraints, life-cycle economic evaluation, and regulatory requirements. By synthesizing recent advances in reactor design, electrode materials, plasma discharge control, and system integration, the review identifies emerging research trends and unresolved technical challenges that influence the future development of ozone technologies.^14^ Overall, by integrating mechanistic understanding with system-level analysis and application-oriented evaluation, this review provides a comprehensive perspective on the development and deployment of ozone generation technologies and highlights key opportunities for improving the efficiency, safety, and sustainability of ozone-based environmental treatment processes.

## 2. Operational properties and safety assessment of ozone in environmental systems

### 2.1. Fundamental physical and chemical characteristics of ozone

**Fig. 2:**
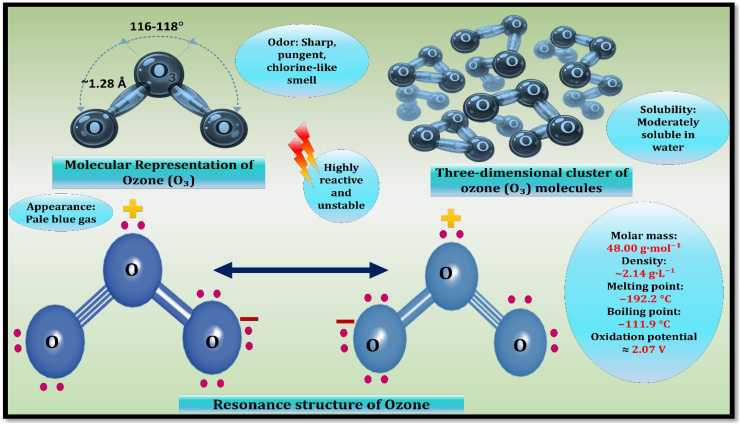
Stoichiometric composition and molecular formula of ozone. Reproduced with permission from Elsevier, 2018.^15^ Copyright © 2018 Elsevier. License number: 6250821175568.

Ozone (O_3_) is a triatomic allotrope of oxygen of wide application in environmental science, industrial processing, and biology. At normal temperature and pressure, it is a weak blue gas and takes a non-linear molecular structure with a bond angle near 116.70 (Fig. 2). The oxidative strength of ozone is the determinant in its practical use, but the same reactivity causes a lack of chemical stability that limits its storage and long-range transportation.^16^ Consequently, a thorough understanding of the physicochemical behavior of ozone is essential in the optimization of the processes of generating ozone, the expansion of the utility of ozone as well as the instrumentation of controlled and dependable functioning in real systems.

#### 2.1.1. Physical characteristics governing ozone behavior

Ozone exhibits high levels of appearance differences with regard to its physical condition. It turns to the faint tint of bluish in the gas phase, and liquefied ozone is bluish with an intense color and the solid form is characterized by a dark violet hue.^17^ Such visual properties are based on the selective absorption of the processes of the electronic transitions that occur in the ultraviolet and visible ranges, especially in those connected with the Hartley and Chappuis bands.^18^ However, at environmentally significant concentrations, gaseous ozone is practically colourless and is not easy to see with the naked eye. Compared to oxygen, the ozone has a significantly greater affinity towards dissolution in water and the coefficient of solubility under normal conditions is about 0.494. This is a property that facilitates effective gas–liquid exchange in aqueous treatment.^19^

Persistence of ozone when added to water is regulated by a combination of interacting variables that may be divided into ambient conditions, chemical composition of the medium, and system operation.^20^ The major environmental factors are temperature, pH and pressure, whereas chemical reactivity is controlled by the dissolved salts, transition metals and organic compounds. Moreover, the method of ozone production, contact strategy, and handling practices are critically important factors in the effective lifetime.^21^ Ozone breaks down quickly, and in most cases, in a matter of minutes, under high-temperature conditions or conditions with strong acidic or alkaline pH, or when reactive solutes are in high concentrations.^22^ Conversely, high-purity water, low temperatures and near-neutral pH can greatly stabilize ozone and it can last hours or longer. The effects of environmental and compositional parameters are often non-linear and system dependent unlike the operational factors which are usually predictable.

Thermodynamically, the melting points of ozone are −192 °C and boiling points −111 °C. It is heavier than air by approximately 1.66 times as it is gaseous, with a density of 2.14 g L^−1^ (ref. 23) even though it is possible to condense to liquid or solid phases in the conditions of cryogenicity, these phases are inherently metastable. Ozone is dangerous at higher than 1011 vol% of concentration at ambient temperature and atmospheric pressure on the back of quick chain degradation resulting in full conversion to oxygen and in strongly confined systems, explosive behaviour.^24^ This deliverability practically excludes the traditional storage and the transportation over long distances. Although ozone dissolution in water is nominal to the Henry law, the actual mass transfer is highly dependent on the hydrodynamic conditions, water chemistry, and conform reaction consumption.^25,26^ Ozone is therefore normally produced on the ground and used instantly as a measure of ensuring effectiveness and safety.

#### 2.1.2. Reaction mechanisms and chemical reactivity of ozone

Ozone is an unusually strong oxidizing agent and this is demonstrated by its high standard redox potential of approximately +2.07 V.^27^ This value presents ozone as one of the strongest oxidants that are normally found, only second to fluorine and stronger than most of the traditional oxidizing agents, as summarized in Table 1. The ozone in the environment is thermodynamically unstable and breaks up easily in a network of complex and interrelated reaction mechanisms.

1 2O_3_ → 3O^2^ Δ*H* = −285 kJ mol^−1^

**Table 1:** A comparative thermodynamic analysis of redox potentials in high-strength oxidizing agents^28,29^

S. no.	Oxidant	Electrochemical reaction	Standard electrode potential *E*° (V)
1	Fluorine	F_2_ + 2e^−^ → 2F^−^	2.87
2	Ozone (acidic medium)	O_3_ + 2H^+^ + 2e^−^ → O_2_ + H_2_O	2.07
3	Hydrogen peroxide (acidic)	H_2_O_2_ + 2H^+^ + 2e^−^ → 2H_2_O	1.78
4	Permanganate (acidic)	MnO_4_^−^ + 8H^+^ + 5e^−^ → Mn^2+^ + 4H_2_O	1.51
5	Chlorine	Cl_2_ + 2e^−^ → 2Cl^−^	1.36
6	Chromium(vi)	Cr_2_O_7_^2−^ + 14H^+^ + 6e^−^ → 2Cr^3+^ + 7H_2_O	1.33
7	Oxygen	O_2_ + 4H^+^ + 4e^−^ → 2H_2_O	1.23
8	Bromine	Br_2_ + 2e^−^ → 2Br^−^	1.09
9	Nitric acid	NO_3_^−^ + 4H^+^ + 3e^−^ → NO + 2H_2_O	0.96
10	Ferric ion	Fe^3+^ + e^−^ → Fe^2+^	0.77
11	Iodine	I_2_ + 2e^−^ → 2I^−^	0.54
12	Copper(ii) ion	Cu^2+^ + 2e^−^ → Cu	0.34

At ambient conditions, ozone is not very unstable but its thermal decomposition rate is extremely high as temperature rises. At temperatures above about 200 °C, the process of ozone degradation sets in, and above 270 °C almost immediately the ozone starts breaking down.^30^ Oxidation in aqueous systems is facilitated by ozone through both direct molecular reactions and self-decomposition pathways that generate highly reactive intermediates, particularly hydroxyl radicals (˙OH), which play a critical role in advanced oxidation processes.^31^ Other radical-based processes can be formed in the gas phase as well, particularly in high relative humidity conditions where the presence of water vapor enhances the formation and propagation of radicals.

2 O_3_ + OH^−^ → OH_2_^−^ + O_2_

3 O_3_ + HO_2_^−^ → ˙OH + O_2_˙^−^ + O_2_

The dissolution of ozone in water is much faster than at room temperature and its lifetime is highly influenced by the quality of the aquatic environment. Ozone may remain longer in highly purified water with half-lives of several hours or longer.^32^ This greater stability is principally associated with reduced temperatures and the lack of reactive species, but the actions of physical processes of dissolving gases may also affect ozone behavior.^33^ As an illustration, gas microbubbles may cause the increase of ozone loss: since such bubbles ascend through the liquid and burst at the surface, the ozone will escape and break at the rate, which essentially diminishes its overall stability. Further, ozone is a very reactive oxidant and can easily react with a wide variety of dissolved inorganic and organic molecules and this once again leads to its rapid depletion in water bodies.^34^

Ozone is considered one of the strongest oxidizing agents that can provoke the emergence of a great diversity of chemical and biological changes. It has an easy time converting inorganic species like ferrous iron, manganous ions, and sulfide compounds in a water treatment system into more resistant oxidized states, thereby enhancing their cleaning ability.^35^ The ozone is also involved in reactions with organic compounds, especially unsaturated bonds and results to cleavage of the molecules, producing lower molecular weight products; aldehydes, ketones, and carboxylic acids.^36^ These reactions also play important roles in the degradation of pollutants and in selective chemical synthesis reactions.^37^ The antimicrobial effects of ozone in biological systems are severe, rupturing cellular membranes and oxidizing vital intracellular constituents which ultimately cause irreversible damage and loss of microorganism viability.^38^

Simultaneously, ozone has functional benefits and limitations.^39^ It is a strong oxidant with good solubility in water and the ability of both primary and secondary radical reaction, which makes it very effective in environmental cleanup, disinfection, and chemical processes that are environmentally friendly.^40^ Nevertheless, ozone is fundamentally volatile and decays quickly, so it cannot be stored for a long time and causes issues with the control of the operation, its safety, and integration into the system. Such balance of reactivity and instability appreciation is then necessary to the optimized design, safe operation, and further development of ozone-based technologies 2.2. Ozone measurement and analytical evaluation.^41^

#### 2.1.3. Monitoring and evaluation metrics

It is crucial to evaluate the performance of the ozone generation systems in order to make sure that they are used in environmental engineering, in healthcare and in industrial practice in a reasonable and safe way. The performance of the systems has a powerful influence on the results of the treatment, the energy consumption, spending on the work, and the overall economic feasibility of the ozone based treatment.^42^ Ozone systems are typically evaluated based on a combination of parameters of output, efficiency, and safety, as indicated in Table 2. These indicators are: the concentration of the ozone, the capacity to produce ozone, the productivity, the consumption of specific energy (SEC), and the adherence to safety standards. When these parameters are taken into account, they will present a strong foundation to contrast technologies and optimize working conditions.^43^

**Table 2:** Process optimization metrics for advanced ozone generation systems

S. no.	Metric	Definition	Formula & unit	Advantages	Limitations	Ref.
1	Ozone concentration	Amount of ozone present in the output gas or liquid stream	*C*_O_3__ = *V*_*m*O_3__/*V*, unit: g m^−3^, mg L^−1^, or ppm	Simple to measure; indicates generator capability	Does not reflect production rate or energy efficiency	7
2	Ozone production rate	Mass of ozone produced per unit time	PO_3_ = *C*_O_3__ × *Q*, unit: g h^−1^	Useful for capacity comparison	Depends strongly on flow rate and operating conditions	13
3	Specific energy consumption (SEC)	Electrical energy required to produce unit mass of ozone	SEC = *P*_O_3__/*P*_elec_, unit: kWh kg^−1^	Key indicator of energy efficiency	Sensitive to measurement errors and system losses	17
4	Energy yield	Ozone produced per unit electrical energy input	*η* = Δ*H*_O_3__/*P*_elec_ Δ*H*_O_3__ × 100, unit: g kWh^−1^	Widely used benchmark; easy comparison across systems	Inverse relationship with SEC may cause confusion	33
5	Ozone generation efficiency	Fraction of input electrical energy converted into ozone formation	*η*_u_ = O_3_ generated/O_3_ used × 100, unit: %	Reflects thermodynamic performance	Requires accurate enthalpy values; idealized metric	54
6	Ozone utilization efficiency	Fraction of generated ozone actually consumed in the target process	*η*_u_ = O_3_ generated/O_3_ used × 100, unit: %	Indicates process effectiveness	Difficult to quantify ozone losses precisely	59
7	Ozone transfer efficiency	Portion of ozone transferred from gas phase to liquid phase	*η*_*t*_ = O_3_ absorbed/O_3_ supplied × 100, unit: %	Critical for water and wastewater treatment	Strongly affected by hydrodynamics and reactor design	61
8	Ozone decomposition rate	Rate at which ozone decays due to thermal or catalytic effects	*K*_d_ = −*d*_CO_3__/*d*_*t*_, unit: s^−1^	Helps evaluate stability and reactor losses	Requires time-resolved measurements	75
9	Current efficiency (electrolytic systems)	Fraction of electrical current contributing to ozone formation	CE = *nFP*_O_3__/*I* ×100. Unit: %	Essential for electrolytic ozone generators	Not applicable to UV or DBD systems	76
10	Process performance index (PPI)	Combined indicator of ozone yield, energy use, and treatment effect	PPI = treatment efficiency/SEC. Unit: dimensionless	Enables holistic system comparison	Lacks standard definition across studies	81
11	By-product formation index	Extent of undesired by-products (*e.g.*, NO_*x*_, bromate)	Ratio-based or concentration-based, unit: mg L^−1^ or dimensionless	Important for safety and compliance	Analytical complexity; system-specific	85

A primary measure of oxidative power and dose efficacy is the concentration of ozone. It can be either in the form of a mass based or a volumetric fraction, depending on the use. Mass concentration is typically favored in water treatment and volumetric concentration is more frequently employed to specify technical grades of ozone in the gas phase.^44^ Ozone generation rate or the rate of production of ozone per unit of time is used to denote scale and throughput of the equipment. To be compared meaningfully, this parameter will have to be reported in addition to the flow rate of feed gas and operating conditions.^45^

The efficiency of gas utilization is measured in ozone productivity that indicates the relationship between the output of the ozone and the quantity of the feed gas that is provided. Recently, the productivity values achieved by the use of dielectric barrier discharge (DBD) technology showed a considerable increase to the theoretical maximum of about 200 g m^−1^ with the recent developments, reflecting the substantial progress in the conversion efficiency.^46^ Specific energy consumption is often used to measure energy performance, which is the electrical energy that is needed to produce one-unit mass of ozone. Less SEC values signify a more efficient work, and oxygen-fed DBD systems tend to have better performance than air-fed or ultraviolet-based generators.^47,48^ Power supply efficiency, reactor configuration, a choice of a dielectric material, thermal regulation, and feed gas purity are some of the factors that affect SEC. Other than performance and efficiency, safety considerations are central to the evaluation of the ozone systems.^49^ Ozone is a very reactive oxidant which may be harmful to health in cases where it has surpassed its recommended levels, especially to the respiratory system. This is why the operational ozone concentrations should be well monitored and controlled as prescribed by the occupational safety regulations. Adherence to these limits is not only necessary to ensure personnel protection but also to direct system design and control measures that assure a safe and stable system operation.^50^

#### 2.1.4. Experimental measurement techniques

The precision to measure the ozone concentration is the core of both research and industrial practices and relies upon the application of appropriate methods of analysis with their respective measurement principles and practical constraints.^51,52^ Ozone can exist in gaseous and dissolved form, therefore, the detection methods applied depend greatly on the state of investigation. Table 3 thus summarizes the most widely used ozone monitoring methods, their corresponding application phases and the safety and certification concerns related to these methods.

**Table 3:** Conceptual framework for ozone detection across environmental matrices

Method	Phase	Principle	Advantages	Limitations	Applications	Safety & certification requirements
UV absorption (254 nm)	Gas	Ozone strongly absorbs UV light at 254 nm; absorbance is proportional to concentration (Beer–Lambert law)	High accuracy, real-time, reference method	High cost, sensitive to dust and humidity	Ambient air: ozone monitoring for environmental air quality	Calibration: use certified ozone sources for accurate and traceable calibration
Electrochemical sensor	Gas	Ozone undergoes redox reaction at electrode producing measurable current	Portable, low cost, low power	Cross-sensitivity to NO_2_/Cl_2_, limited lifespan	Workplace safety monitors, indoor air quality	Periodic calibration; certified for occupational exposure limits (OSHA/NIOSH)
Chemiluminescence	Gas	Ozone reacts with ethylene or nitric oxide producing light proportional to concentration	Very high sensitivity, fast response	Expensive, complex operation	Atmospheric research, trace-level ozone detection	Proper handling of reactant gases; laboratory safety compliance
Iodometric titration	Gas/liquid	Ozone oxidizes iodide to iodine, which is titrated quantitatively	Simple, accurate, classical method	Time-consuming, not real-time	Calibration of ozone generators, laboratory analysis	Chemical handling safety; standard analytical lab certification
Indigo trisulfonate method	Liquid	Ozone decolorizes indigo dye; color loss measured spectrophotometric ally	Selective, suitable for aqueous ozone	Interference from strong oxidants	Water treatment plants, drinking water analysis	Use of certified reagents; compliance with APHA/ISO water testing standards
Colorimetric detector tubes	Gas	Ozone reacts with chemical reagent causing visible color change	Simple, low cost, no power needed	Semi-quantitative, single-use	Quick field checks, emergency response	Tubes certified by manufacturer; PPE required during sampling
Semiconductor (metal oxide) sensor	Gas	Ozone changes electrical resistance of heated metal oxide surface	Low cost, compact	Poor selectivity, affected by humidity	Consumer air monitors, preliminary screening	Electrical safety compliance; not suitable for regulatory use
Fluorescence method	Gas	UV-excited ozone emits fluorescence proportional to concentration	High sensitivity, rapid response	Instrument complexity, high cost	Advanced atmospheric studies	UV safety compliance; periodic optical calibration
Potassium permanganate method	Gas	Ozone oxidizes KMnO_4_ leading to color change	Simple qualitative indication	Low sensitivity, interference issues	Educational demonstrations, rough estimation	Chemical handling standards; not for precision monitoring
Gas chromatography (GC)	Gas	Ozone detected indirectly *via* decomposition products	High specificity	Complex, not real-time	Research laboratories	Certified analytical labs; strict ozone destruction before GC inlet

(1) Iodometric titration is widely used as a reference method for quantitative determination of dissolved ozone in aqueous solutions because ozone oxidizes iodide ions to iodine under acidic or neutral conditions. The generated iodine is titrated with standardized sodium thiosulfate, allowing ozone concentration to be determined from the reaction stoichiometry.^53^

4 O_3_ + 2KI + H_2_O → I_2_ + O_2_ + 2KOH

Iodometric titration is commonly applied as a reference method of laboratory calibration of dissolved ozone measurements due to its accuracy and low costs of operation. Nevertheless, interference by other oxidizing agents affects the method, and the off-line analysis is tedious, and, therefore, limits the use of the technique to continuous or *in situ* monitoring.

(2) The ultraviolet absorption spectroscopy is mainly used in quantifying the ozone in the gaseous state. It is based on the high absorption property of ozone at about 254 nm, which makes it possible to determine the concentration according to the Beer Lambert principle.^54^ Owing to its rapid response to signals, dependable operation, and reduced sensitivity to most of the gases that coexist, this method has been the default mode of the continued monitoring in industrial ozone generation and also in the atmospheric measurements. Its application is however hampered by the cost of instrumentation, which is high, the need to have the instrumentation regularly calibrated to ensure that accuracy is maintained as well as gradual loss of the signal due to polluted optical windows during prolonged use.

(3) Sodium indigo disulfonate technique is mainly used in the determination of dissolved ozone in an aqueous solution. In this method, the indigo dye is reacted specifically with ozone, and the color intensity is reduced, which is determined by the absorbance at a wavelength of about 600 nm. This technique has a high sensitivity and less interference by chloride ions in comparison to iodometric titration. However, it cannot be used to monitor continuously or in real time due to its reliance on hand sampling and off-line analysis. It is therefore primarily applied in the laboratory investigation that is related to the treatment processes and the determination of water quality in the environment.^55^

(4) Electrochemical ozone sensors are generally designed to detect ozone in gases. The principle of their detection is the electrochemical reduction of ozone at the working electrode that produces a current response that rises as the concentration of ozone rises. These sensors are common in portable monitoring system and in other work place safety systems due to their small size, relatively low cost and capability to give continuous measurements. Although this has its benefits, the electrochemical sensor is usually affected by practical limitations. Specifically, they are not selective enough and can react to other oxidizing gases like chlorine and nitrogen dioxide. Besides that, they are not as effective in the long-term performance due to signal instability with time, the necessity to make regular calibrations, and the degradation of electrolytes.

(5) An alternative popular method of gaseous ozone monitoring is metal oxide semiconductor sensors. In such sensors, ozone reacts with the surface of the metal oxide material which causes a variation in electrical resistance as a result of variations in the surface charge states. Their basic design, low-cost of manufacture and ability to fit with compact electronic systems have resulted in extensive application to domestic air-quality systems and in low-cost portable detectors. Nonetheless, sensing response is highly influenced by ambient temperature and humidity and such sensing devices do not have the much needed selectivity to ozone. They are therefore normally limited to qualitative or semi-qualitative monitoring and not accurate determination of the ozone concentration.^56^

### 2.2. Safety-related properties and engineering constraints of ozone

Although the effectiveness of ozone as an effective oxidant has long been established, the practical application of ozone is inherently limited by its natural safety and physicochemical constraints and is confined by its operating conditions.^57^ As an occupational health hazard, ozone is known to be a respiratory pollutant and is thus highly regulated with the help of exposure limits in the form of permissible exposure limits and immediately dangerous to life and health values.^58^ Regulatory agencies across different regions have established quantitative exposure limits for ozone in order to protect both occupational workers and the general public. Although these standards are based on similar toxicological evidence, the allowable exposure limits vary slightly depending on the regulatory framework and the averaging period used. For example, the World Health Organization (WHO) recommends an ambient air guideline value of 100 µg m^−3^ for an 8-hour average exposure, while the United States Environmental Protection Agency (US EPA) has established a National Ambient Air Quality Standard of 0.070 ppm averaged over 8 hours. Similarly, the European Union Air Quality Directive specifies a target value of 120 µg m^−3^ for the maximum daily 8-hour mean ozone concentration. In occupational environments, agencies such as the Occupational Safety and Health Administration (OSHA) specify permissible exposure limits to protect workers involved in ozone generation and handling processes. These regional regulatory frameworks highlight the importance of continuous ozone monitoring, proper ventilation systems, and engineering safety controls in industrial and environmental ozone applications.^58^

The exposure to low doses in the short term can irritate the respiratory system and cause inflammatory reactions, whereas exposure to larger doses or unintentional leakage of high doses of the chemical may result in serious pulmonary damage and permanent respiratory disorders.^59^ These hazards require rigorous engineering measures in ozone-related systems such as closed reactor designs, continuous concentration measurements, programmable shutdown systems and built-in ventilation protection measures to avoid accidental human exposures.

Along with its toxicological risks, ozone is also described as highly thermodynamically unstable. The molecule is readily broken down by both reactions in the gas-phase and on surfaces, and a faster rate of decomposition is observed at higher temperatures, higher pressures, or on reactive or catalytic surfaces.^60^ This instability does not allow long-term storage and transportation and demands the production of the ozone on the spot and consumed instantly, which has a potent impact on the system layout and operational strategy. In the case of confined conditions or insufficient ventilation, the excessive accumulation of ozone can lead to a rapid increase in the process of decomposition, which can cause the appearance of local sources of heat, pressure changes, or sudden loss of concentration, which in turn can cause new safety issues.^61^ This has led to a general design of the industrial ozone system to be allowed to run below specific concentration limits to maintain steady and safe operation.

The compatibility of materials also restricts the reliability and life span of the ozone systems. Owing to its high electrophilicity, ozone can easily react with unsaturated bonds and other chemically sensitive functional groups found in most polymers and elastomers, resulting in fast aging process, embrittlement, and mechanical breakdown.^62^ Most engineering materials, including natural rubber, nitrile rubber, and some plastic components, are not very resistant to the influence of the ozone, but only a few materials, including specific fluoropolymers, corrosion-resistant stainless steels, and ceramic materials, show sufficient long-term stability under ozone exposure.^63^ Poor choice of material might lead to leakage and seal degradation, sensor malfunction, and loss of integrity of the system, which have a direct impact on the operational safety and service life. Combined, these health-related, thermodynamic, and materials-based constraints mean that the process of ozone application is not only limited by its ability to oxidize but also necessitates the need to balance between performance and safety and engineering feasibility.^64^ These restrictions are inherent to the ozone itself and not related to a specific use, and ozone generation and handling technologies are at the center of attention in the attempt to control chemical threats with the help of proper electrical, mechanical, and operational design.

## 3. Ozone generation systems: performance and economic analysis

An ozone generator is a technical device that has been established to synthesize ozone artificially by changing oxygen molecules into ozone by using controlled physical or electrochemical methods. Practically, the ozone production techniques are widely classified into three broad categories, namely, the ultraviolet-based generation systems, the dielectric discharge systems based on the dielectric barrier effect, and the electrochemical generation systems. The ultraviolet and dielectric barrier discharge methods can primarily be used to generate ozone in the gaseous form, and subsequently move it into the water or other reaction conditions based on the needs of the operation. In other designs, ultra-violet lamps can be immersed in water, and ozone can be generated in the liquid phase itself. Electrochemical generation of ozone Compared to these methods, electrochemical generation of ozone enables ozone to be generated as a gas or as dissolved ozone in water, and is more flexible in a wide range of uses.^65^

Although these ozone generation technologies share the common objective of producing ozone efficiently, their operational characteristics, energy requirements, and scalability differ significantly. Ultraviolet (UV) ozone generators are generally characterized by simple reactor design and low capital investment; however, their ozone production rate and energy efficiency are relatively limited. Consequently, UV-based systems are primarily suitable for small-scale applications such as laboratory disinfection or localized air purification.^66,72^

In contrast, dielectric barrier discharge (DBD) systems represent the most widely adopted industrial technology for ozone production. These plasma-based systems can achieve significantly higher ozone concentrations and improved energy efficiency compared with UV systems. Nevertheless, DBD generators require high-voltage power supplies and efficient thermal management to maintain stable discharge conditions, which increases system complexity and operational cost.^80,84,106^

Electrochemical ozone generation offers a fundamentally different approach, producing ozone directly in aqueous solution through anodic oxidation reactions. This configuration enables the production of high-purity dissolved ozone without gas–liquid transfer limitations. However, challenges related to electrode stability, material cost, and energy consumption currently limit its large-scale industrial deployment.^107,127^

### 3.1. Photochemical ozone generation using UV radiation

#### 3.1.1. Reaction mechanism and system structure

**Fig. 3:**
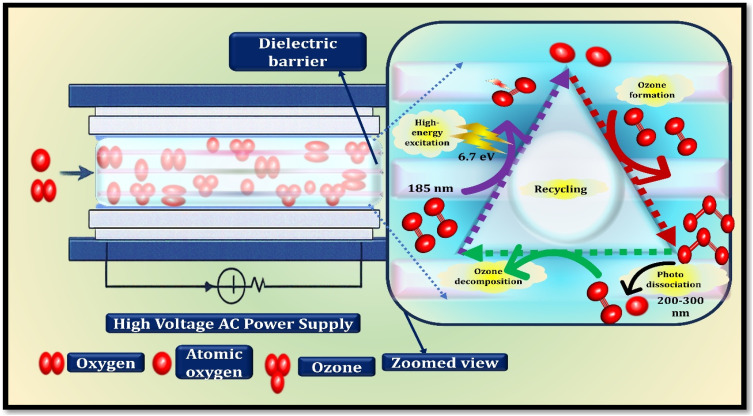
Photochemical ozone generation under UV irradiation: mechanisms and kinetics.

The ultraviolet ozone production is an artificial way of obtaining ozone based on the principles of photochemical reactions.^66^ It is done by exposing molecular oxygen to ultraviolet radiation of suitable wavelengths, and in this manner the process causes reactions similar to those taking place in the natural atmosphere to form ozone. The vacuum ultraviolet radiation in mercury-lamp-based UV ozone generators is the starting point of ozone in the form of photons with an energy of about 6.7 eV and a wavelength of about 185 nm. When oxygen molecules absorb this radiation, O–O bond is broken apart leading to photo dissociation process in which two highly reactive atomic oxygen species are formed (Fig. 3).^67^ These are the oxygen atoms which are then involved in downstream reactions that result into the production of ozone. The basic reaction mechanism is as follows:

5 O_2_ + hv (*λ* = 185 nm) → 2O˙

Newly made oxygen atoms react with molecular oxygen around them. Nonetheless, the reaction is only able to take place efficiently in the presence of a third collision partner (M), typically N_2_ or O_2_, which takes away the surplus energy emitted when forming the bond. The third body cools off this energy and enables the formation of ozone.

6 O˙ + O_2_ + M → O_3_ + M

At the same time, ozone is oxidized in parallel decomposition routes in the system, and reactions of atomic oxygen are the most common quenching reaction.

7 O_3_ + O˙ → 2O_2_

Also, ozone can be destroyed simultaneously in some wavelength beds by ultraviolet-based ozone generation. To a large extent, this effect depends on the spectral output of the UV source, such as whether the emission is narrowband or broadband, and the ratio of the contribution of the vacuum-UV radiation at 185 nm to the longer wavelengths in the 200–300 nm area. The photons at 185 nm enhance the dissociation of oxygen and its further reaction to produce ozone, but ozone is a strong absorber in the wavelength of 200–300 nm, where photochemical dissociation becomes eminent.^68^

8 O_2_ + e^−^ (high energy electrons) → 2O + e^−^ (low energy electrons)

The concentration of ozone within the reactor is regulated in UV-based ozone generation by balancing between the processes of ozone formation and simultaneous degradation of ozone, which occur. In order to enhance the total ozone yield, mercury lamps of practical use are typically constructed with a smaller or no emission of 185 nm that inhibits ozone breakdown by irradiation. Since no high-voltage electric discharges or chemical reagents are used in this method, and only ultraviolet radiation is required, it can be considered to be a clean and technically straightforward process to ozone. An internal fan introduces ambient air into the reaction chamber on a continuous basis during operation.^69^ Under the UV irradiation range, photons at 185 nm are taken in by oxygen molecules and dissociate to form atomic oxygen. These reactive oxygen species then react with the oxygen to produce ozone. The ozone-containing gas produced is also directly generated using air and does not require any external supply of oxygen as compared to the ozone formation and the process does not produce nitrogen oxide by-products. These advantages perform well in UV ozone generators to be used indoors. As illustrated in Fig. 3, a standard UV ozone generator has three components that are critical.^70^

• Three basic functional components usually constitute UV-based ozone generation systems. The main constituent is a low-pressure mercury vapor lamp that is enclosed inside a quartz sleeve that allows the short-wavelength ultraviolet radiation to pass through it. Even though the most active emission is at 253.7 nm, a lower proportion of the radiation at 185 nm is also obtained. It is this wavelength that causes photo dissociation of molecular oxygen resulting in the formation of ozone.

• The photochemical reactions are carried out in a closed reaction chamber consisting of solid material with high ozone corrosion resistance, *e.g.* stainless steel or specially-designed polymers. The chamber has well-considered gas inlet and outlet ports that regulate the flow patterns and residence time hence affecting the efficiency of generation of ozone.

• Electronic power supply with high frequency ballast is used to operate the lamps. This unit maintains constant electrical supply and fills the fluctuations in operation to maintain constant ultraviolet output and constant ozone generation in varying environmental conditions.

Although UV ozone generators are simple to use, there are a number of safety and reliability issues associated with their use. The mercury-containing lamps could be associated with potential risks associated with breakage, dealing with, and disposal. Operation or maintenance may have further safety issues because of exposure to ultraviolet radiation. Furthermore, the aging of a lamp, degradation of ballasts and foul quartz sleeve can lead to variations or losses in ozone. These constraints augment operational maintenance and limit the operational stability of UV based-ozone generation systems in the long run.^71^

#### 3.1.2. An overview of research progress in UV ozone technology

Surface disinfection, treatment of materials, and purification of the environment are some of the activities of UV ozone technology that have garnered progressively growing interest due to its efficiency, environmental friendliness, and ease of operation. Though past research has been conducted on the various applications, they all revolve around the same principle, which is the joint effect of ultraviolet radiation and ozone. In the case of the usage of UV light or ozone, efficacies of disinfection are frequently restricted by inefficient penetration, lack of uniform exposure, or reaction pathways as stated by Criscuolo *et al.*^72^ Conversely, UV/ozone treatment simultaneously avoids these limitations by making it able to cover a wider area and greater oxidation presence of the radicals, resulting in more even and effective pathogen inactivation. The current photochemical and oxidative mechanism is contrasted with single-method disinfection methods and pits the first against the second.

The usefulness of UV-ozone treatment has been verified by a number of studies that have been carried out to clean surfaces and alter materials. The reactive ozone species interact with surface layers with very shallow depths, and organic contaminants can be eliminated without altering bulk material properties. The effectiveness of UV-ozone treatment has been demonstrated in several studies involving surface cleaning and material modification. Reactive ozone species generated under UV irradiation interact with surface contaminants and promote oxidation reactions that remove organic residues without significantly altering bulk material properties. For example, UV-ozone treatment of TiO_2_ surfaces has been shown to effectively remove organic contaminants while simultaneously improving surface wettability and increasing the density of hydroxyl groups on the surface. These modifications enhance interfacial charge transfer processes and can significantly improve the performance of photovoltaic and photocatalytic systems, with reported improvements in conversion efficiency from approximately 1.43 to 2.53 in certain experimental studies.^73^ These modifications enhance charge transfer at interfaces leading to a significant increase in photovoltaic performance, and the conversion efficiency improved to 2.53, as compared to 1.43. This should indicate that UV ozone treatment can be a good low-temperature surface modification technique as opposed to a mere cleaning procedure. A study by Khuntia *et al.*^74^ on the removal of SO_2_ and NO_8_ also suggests that a high removal efficiency can be attained with a relatively low ozone production and a moderate UV wavelength. This observation indicates that reaction pathways, radical formation efficiency, and mass transport are important in performance of the processes than the concentration of ozone itself. The latest studies also emphasize on the significance of system integration. According to automated UV-ozone systems will save on the time of treatment and variability in the operation of the system and other materials that are likely to lose their properties. This must be improved in order to be reliable on a large scale. Moreover, the UV-C-ozone air-purification plan has expanded the scope of the UV-ozone strategy to controlled indoor space applications and can be used in air disinfection, indoor air quality management, and smart building solutions.^75^

#### 3.1.3. Performance advantages and practical challenges of UV ozone

UV-based ozone generation systems offer several practical advantages including simple reactor design, relatively low capital cost, and the absence of high-voltage electrical components required in plasma-based systems. However, their ozone production rate and energy efficiency are generally lower than those of dielectric barrier discharge systems, which limits their application in large-scale industrial processes. Though past research has been conducted on the various applications, they all revolve around the same principle, which is the joint effect of ultraviolet radiation and ozone. In the case of the usage of UV light or ozone, efficacies of disinfection are frequently restricted by inefficient penetration, lack of uniform exposure, or reaction pathways as stated.^76^ Conversely, UV/ozone treatment simultaneously avoids these limitations by making it able to cover a wider area and greater oxidation presence of the radicals, resulting in more even and effective pathogen inactivation. The current photochemical and oxidative mechanism is contrasted with single-method disinfection methods and pits the first against the second.

UV-based ozone generation systems offer several practical advantages including simple reactor design, relatively low capital cost, and the absence of high-voltage electrical components required in plasma-based systems. These characteristics make UV ozone generators suitable for small-scale disinfection, surface treatment, and indoor air purification applications. However, their ozone production rate and energy efficiency are generally lower than those of dielectric barrier discharge systems, which limits their use in large-scale industrial processes. In addition, UV lamp aging, quartz sleeve fouling, and fluctuations in ultraviolet intensity can gradually reduce ozone production efficiency and require periodic maintenance and lamp replacement.^71,77^ A study by on the removal of SO_2_ and NO_8_ also suggests that a high removal efficiency can be attained with a relatively low ozone production and a moderate UV wavelength. This observation indicates that reaction pathways, radical formation efficiency, and mass transport are important in performance of the processes than the concentration of ozone itself.^78^ The latest studies also emphasise on the significance of system integration. To automated UV-ozone systems will save on the time of treatment and variability in the operation of the system and other materials that are likely to lose their properties. This must be improved in order to be reliable on a large scale. Moreover, the UV-C-ozone air-purification plan by has expanded the scope of the UV-ozone strategy to controlled indoor space applications and can be used in air disinfection, indoor air quality management, and smart building solutions.^79^

### 3.2. Dielectric barrier discharge-based ozone generation system

#### 3.2.1. Reaction pathways and structural composition of the system

Dielectric barrier discharge (DBD) ozone generation is founded on the establishment of non-thermal atmospheric pressure plasma.^80^ In a standard DBD reactor, the electrodes are spaced by a gas gap and at least one of the electrodes is coated with an insulating dielectric material, *e.g.* glass or ceramic, with a large value of dielectric constant. In cases where large alternating voltages in the kilovolt range are used, the electric field causes a large number of momentary filamentary discharges of short duration across the gap. These micro-discharges should be shorter than nanoseconds to microseconds.^81,82^ The charging up of the dielectric layer on the surface quickly restrains the discharge current and each of the filaments goes off spontaneously. This self-restraint effect causes the arc to be closed and the gas temperature of the plasma to remain low and this condition creates stable operating conditions that are conducive to ozone production.^83^ In such a plasma atmosphere, the energetic electrons interact with oxygen molecules to start the dissociation process of the molecules resulting in the formation of ozone.

9 O + O_2_ + M → O_3_ + M

Oxygen atoms formed are then paired with the molecular oxygen, and a third-body species (M) takes up the surplus energy resulting in the formation of ozone in a three-body reaction.

10 O_3_ + O → 2O_2_

These molecules prove to be extremely significant since they absorb any surplus of energy and in addition, they preserve the momentum that balances the newly formed ozone. Meanwhile, reverse dissociation reaction is used to reduce ozone.

11 O_3_ + e^−^ → O_2_ + O + e^−^

12 3H_2_O → O_3_ + 6H^+^ + 6e^−^ (*E*^a^ = +1.51 V)

**Fig. 4:**
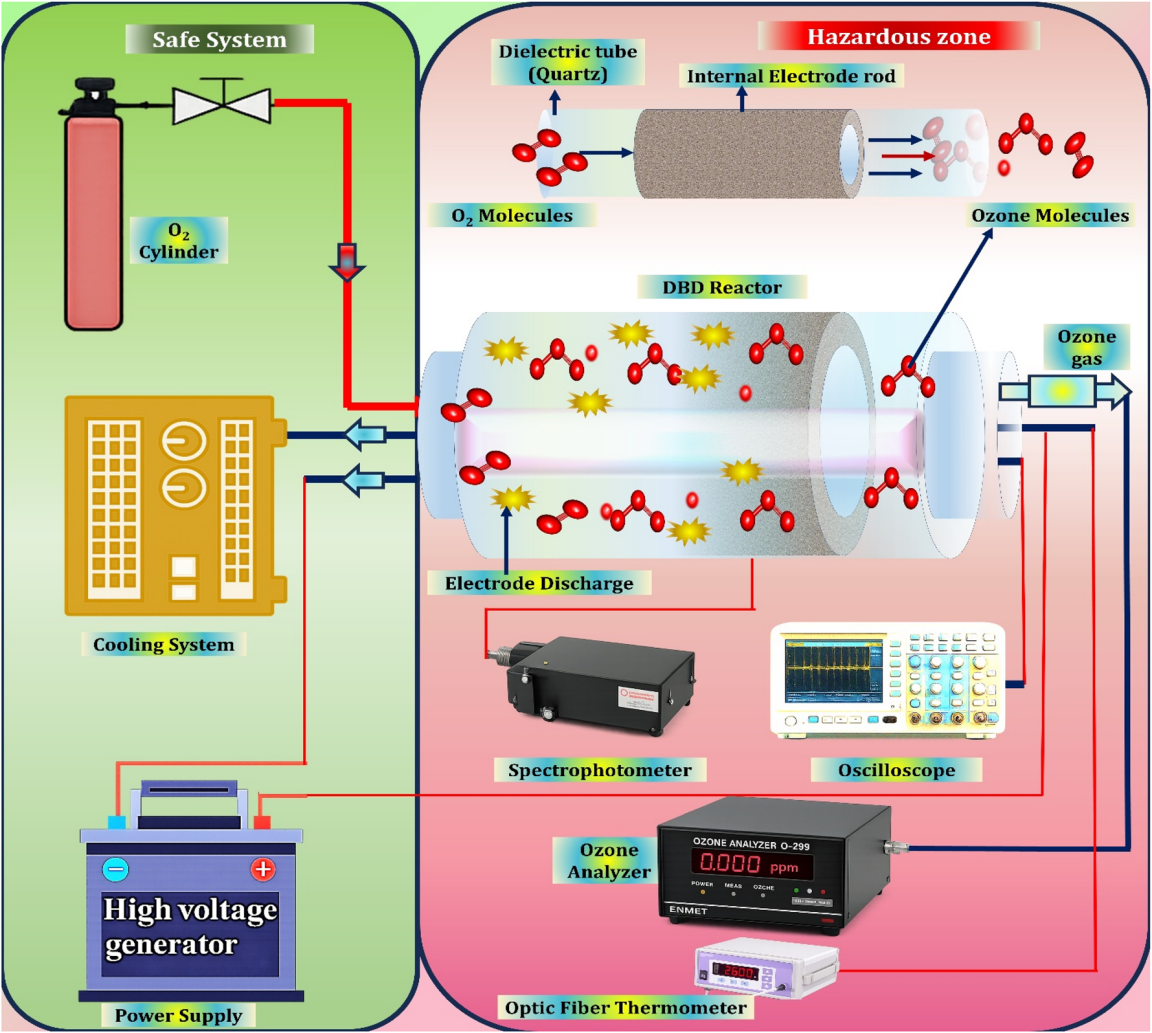
Plasma-driven ozone formation in dielectric barrier discharge systems. A generic dielectric barrier discharge (DBD) ozone production system consists of a sequence of not independent sub systems, each of which plays a crucial role in the establishment of the operation effectiveness, stability, as well as in the establishment of security.

A conventional dielectric barrier discharge (DBD) ozone generator has a working principle shown in Fig. 4 schematically. The rate of effective interacting of excited electrons with oxygen molecules, the ability to maintain appropriate gas temperatures of the discharge, and the stability and consistency of applying electrical field are the key determinants of the creation of ozone in DBD reactors.^84^ These are the considerations that are directly related to each other concerning the efficiency of energy conversion and the system performance as regards to the generation of ozone.

❖ The gas supply and conditioning unit feeds the oxygen of high purity enough to be used as the feed gas in forming of ozone. High-quality oxygen consumption will be an effective way of reducing the production of nitrogen oxides and minimize the possibility of corrosive damage to downstream equipment. Homeostatic gas composition at this level is necessary to ensure that the ozone is produced on a regular basis and the overall system service life.

❖ The reactor with the cooling assembly is the centre of ozone production process. Other widespread reactor designs are coaxial tubular designs, and parallel plate designs, in which the electrodes are physically isolated by a dielectric barrier, relative to the plasma region. Due to the thermo unstable nature of ozone, there is a need to remove the heat effectively. Water circulation is commonly employed as integrated cooling to keep the temperature of discharge at a temperature below 20 °C to inhibit ozone decomposition and permit stable output performance.

❖ The power supply which is of high voltage and high frequency is used to convert the common grid power into electrical power that is used to maintain the discharge. It has a direct effect on the yield of ozone and specific energy consumption, and this makes it a decisive factor in terms of productivity and operating cost. Stability of power supply is especially significant when it comes to preventing changes in ozone concentration as long as the system is running.

❖ Operation in the system is determined by a control and monitoring platform, which is typically founded on programmable logic controllers or embedded control units. This subsystem is constant monitoring of key operating variables as voltage, current, temperature, rate of flowing gas, and pressure. It has closed-loop regulation and safety interlocks, which make it stable and allow automated control under different load conditions.^85^

Though used extensively in industries, DBD ozone generators are inherently unsafe and unreliable. High-voltage components may pose an electrical risk during operation and maintenance, particularly when not properly isolated and locked out. Also, long-term interaction with plasma and thermal stress causes the gradual destruction of the components of critical elements, including dielectric materials and electrodes and, in addition, power supply instability. Such failure modes may cause intermittent ozone production, and in many cases back-up or increased maintenance plans are necessary to be able to guarantee long-term dependability.^86^

#### 3.2.2. Research developments in DBD ozone generation technology

Studies of dielectric barrier discharge (DBD) ozone generators have slowly progressed to what can be termed as a modification of the basic performance optimization of these devices to an in-depth analysis of plasma dynamics, reactor technology, and reliability. Recent research is increasingly appreciating the fact that the productiveness of ozone, energy efficiency, and system durability are not determined by one parameter but a complex combination between discharge properties, electrical stimulation, and reactor design.^87^

The initial studies of discharge behavior have established that the conditions in the plasma are decisive in the formation of ozone. Zhang *et al.*^88^ studied the process of ozone generation in air-fed DBD reactors, and proved that electrical operating conditions as well as ambient humidity has a strong impact on micro-discharge processes and electron energy distributions. Their results showed that moisture changes discharge stability and plasma chemistry thus influencing ozone formation pathways. Although the controlled humidity could enhance the consistency of the operation, a large amount of water vapor encourages the reactions of the ozone destruction process which sets an internal limit of the ozone yield in an air based system.^89^ Together with the plasma studies, much effort has been concentrated on the enhancement of reactor configurations. To a modified DBD reactor, in which a conductive layer of silver was placed between the dielectric barrier and electrode. The result of this structural change was a steadier discharge and a lower degree of localized fila mentation, which produced a steady discharge of ozone. Nevertheless, it has benefits, and the long-term stability, material degradation as well as economic viability of the application of noble metal-layers are still open to questions especially when it comes to large scale or continuous use.^90^

The development of power supply strategies has increased the design space of the DBD ozone generators further. Kim *et al.*^91^ stated that even when the repetition frequencies are over 10 kHz, bipolar pulsed excitation can significantly increase the concentration of ozone. The achievement of this was explained by the fact that intensive, short-lived micro-discharges were formed that promoted the production of ozone and prevented excessive heating of gases. The greater technical complexity and expense of high-frequency pulsed power systems however, may limit their use in industrial applications.

There is also increased attention on scaling and modularization of DBD reactors. In a multi-tube parallel DBD generator was compared with a single tube reactor with the same discharge volume. The multi-tube design was found to reach greater ozone concentrations and was also highly efficient in terms of energy consumption and this was mainly because of the increased area of discharge and the ability to effectively dissipate the heat. Meanwhile, the research also pointed out more practical difficulties in the form of the necessity to achieve consistency in gas flow and electrical loading of parallel channels.^92^ The ozone generation in atmospheric oxygen in various planar DBD discharge modes. their findings indicated that alterations in discharge mode have a substantial influence on the formation of reactive species and the prevailing ozone formation mechanisms. In spite of the fact that these results can be used in a discharge-mode-oriented optimization, the conversion of such control strategies into industrial-scale systems is still technically difficult.^93^

Not so recently, Driss *et al.*^94^ created a new DBD ozone generator with high-voltage electrodes in the form of a series of spheres of stainless steel. This arrangement concentrated ozone in the locality and did not proportionally increase power consumption as it increased the intensity of the electric field.

Although numerous studies have reported improvements in ozone production efficiency through modifications in reactor configuration, dielectric materials, and power supply strategies, direct comparison between these results remains challenging. Differences in experimental conditions such as gas composition, humidity levels, discharge gap distance, and operating frequency can significantly influence plasma characteristics and ozone formation pathways. As a result, ozone yields reported in different studies often vary widely even for similar reactor designs.^88,91^ In addition, some studies report that increasing discharge power and operating frequency enhances ozone generation efficiency, whereas other investigations indicate that excessive power input can increase gas temperature and accelerate ozone decomposition, thereby reducing overall ozone yield.^92,93^ These conflicting findings suggest that optimal operating conditions depend strongly on reactor geometry, feed gas composition, and thermal management strategies. Consequently, future research should focus on establishing standardized experimental methodologies and reporting protocols to enable more reliable comparison of DBD ozone generation performance across different studies. However, the high field non-uniformity of this design could hasten the wear rate of the electrodes and could cast doubt on long term working reliability is exhibited in Table 4.

**Table 4:** Discharge physics and electron energy distribution in DBD-driven ozone synthesis

S. no.	Year	Research content	Ozone production/energy yield (g kW^−1^ h^−1^)	Conclusion	Ref.
1	2000–2003	Basic DBD reactor design using glass/ceramic dielectrics and air feed	20–40	Demonstrated feasibility of DBD for ozone generation; efficiency limited by air humidity and NO_*x*_ formation	95
2	2004–2006	Effect of dielectric material, electrode gap, and AC frequency	40–70	Improved dielectric selection and optimized gap significantly enhanced ozone yield	96
3	2007–2009	Oxygen-fed DBD systems and cooling strategies	80–120	Oxygen feed and effective heat removal markedly increased ozone production efficiency	97
4	2010–2012	Pulsed power supply and micro-discharge control	120–160	Pulsed excitation reduced energy losses and improved plasma uniformity	98
5	2013–2015	Surface-modified electrodes and nanostructured dielectrics	150–200	Enhanced surface area promoted micro-discharges and higher ozone yield	99
6	2016–2018	Multi-stage and modular DBD reactor configurations	180–250	Scale-up possible without severe efficiency loss; suitable for industrial use	100
7	2019–2020	Computational modelling and plasma–chemistry optimization	220–300	Numerical optimization enabled precise control of operating parameters	101
	2021–2022	Integration with advanced cooling and oxygen recycling	250–350	Thermal management became a key factor for sustained high ozone yield	102
8	2007–2009	Oxygen-fed DBD systems and cooling strategies	80–120	Oxygen feed and effective heat removal markedly increased ozone production efficiency	103
9	2023–2024	High-frequency power electronics and AI-assisted control	300–420	Smart control systems significantly improved energy efficiency and operational stability	104
10	2025 (recent)	Hybrid DBD systems with catalyst-assisted ozone stabilization	350–500	Emerging designs show strong potential for ultra-high efficiency and long-term operation	105

On the whole, the recent advances lead to the idea that the further development of DBD ozone generators should rely on the synchronized development of the control of plasmas, electric stimulation and the design of the reactor. Although these successes have been recorded, the need to balance high ozone yield and low energy use with continuous operational stability continues to be a challenge especially in unstable environmental conditions. To solve these problems, there will be the need of combining the design strategies and high level control strategies that will involve the balance of performance and durability and reasonable practicality.

#### 3.2.3. Benefits and challenges of DBD-based ozone generation

The most developed technique of large-scale ozone production is the dielectric barrier discharge (DBD) technology which is extensively used in the treatment of water, oxidation of industries, as well as disinfection processes. High production of ozone with consistent operation particularly when pure oxygen is used has led to long-term application in industry. Nonetheless, high cost of installation and high power consumption are still significant constraints. Operating temperature also affects the performance of systems, where high temperatures cause faster decomposition of ozone and low productivity. These aspects limit the use in energy-sensitive and economically sensitive areas. The present studies are thus aimed at enhancing the efficiency of energy and reliable functioning. The main activities are the improved power supply regulation and the low-loss dielectric materials and better discharge structures in order to increase the use of the plasma. They are also exploring ways of operating with renewable energy and waste heat recovery as a way of reducing operating costs and emissions. Through these advancements, DBD technology should continue being an important part of the design of ozone generation to be applied in the industrial and environmental context.^106^

### 3.3. Electrochemical ozone production for water treatment applications

#### 3.3.1. Process mechanisms and system configuration

Generation of electrolytic ozone depends on the electrochemical oxidation process which occurs at anode. When an external potential is applied, the oxidation of water at the anode surface is encouraged to encourage the generation of the ozone using the high energy anodic reactions instead of the traditional oxygen evolution route.

Anodic ozone formation3H_2_O → O_3_ + 6H^+^ + 6e^−^

Competing oxygen evolution reaction

13 2H_2_O → O_2_ + 4H^+^ + 4e^−^ (*E*^a^ = +1.23 V)

Ozone formation occurs alongside oxygen generation at the anode, where the oxygen evolution reaction proceeds concurrently and competes for the same electrochemical sites.

Cathodic hydrogen evolution

14 2H^+^ + 2e^−^ → H_2_

The undesired oxygen evolution reaction in electrochemical ozone generation is reduced by choosing the anode materials which have intrinsically large oxygen evolution over potentials, with boron-doped diamond (BDD) and lead dioxide (PbO_2_) achieving this most significantly.^107^ These electrodes favour ozone formation due to their slow kinetics of releasing oxygen. Electrochemical cell is usually fitted with a proton exchange membrane (PEM) that is a solid electrolyte to facilitate efficient conduction of protons besides keeping the anodic and cathodic reactions separated.^108^ The anode produces ozone that enters the liquid phase directly, producing very concentrated ozone water and the hydrogen gas is produced at the cathode through the hydrogen evolution reaction.

**Fig. 5:**
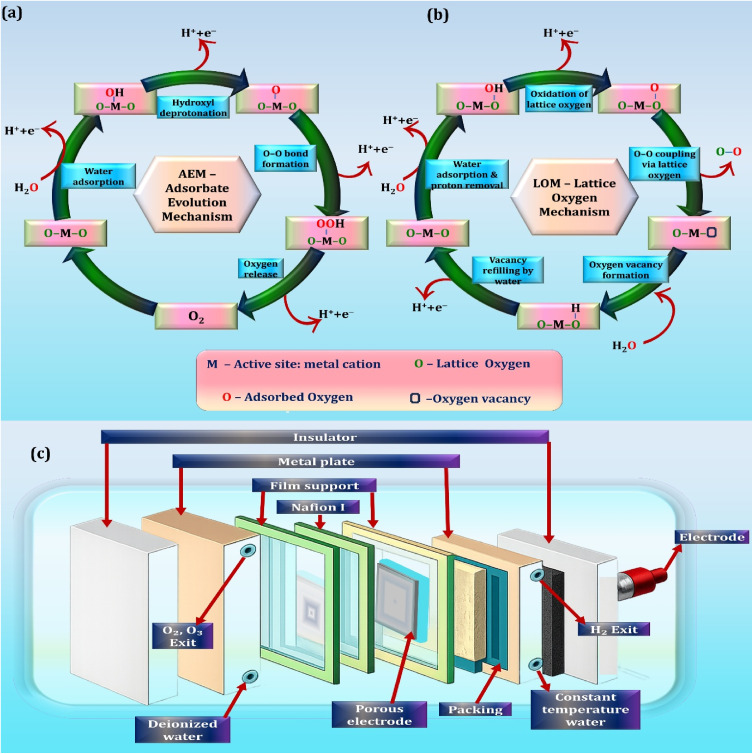
Schematic illustration of (a) the adsorbate evolution mechanism (AEM), (b) the lattice oxygen mechanism (LOM), and (c) an electrolytic ozone generation system.^109^ Reproduced with permission from Elsevier © 2020 License number: 6250790050033.

The schematic diagram in Fig. 5 is a representation of how the electrolytic ozone generator works. Gu *et al.* (2020) state that two four-electron pathways could occur in the process of the formation of ozone at the anode. One route is the ascorbate-emitter route, where oxygen-containing intermediates that adsorb on the electrode surface react sequentially as illustrated in Fig. 5a. The second route is lattice oxygen mediated which involves the direct involvement of lattice oxygen atoms of electrode material in the process of ozone creation; the equivalent reaction cycle is shown in Fig. 5b.

The electrolytic system has a high current efficiency, and the level of purity of the ozone produced is very high. The intrinsic characteristics of the electrode materials, the structural architecture of the ion-exchange membrane, and the operating potential apply to a significant extent to determine its performance. These aspects are optimally reduced to achieve low internal resistance, enhance the use of charge, and to be able to directly generate high-concentration aqueous ozone, thus eliminating the risks of contamination of external ozone gas solutions. Fig. 5c depicts that the electrolytic ozone generator is small and highly integrated, an electrochemical system that consists of a number of functional units that are necessary to ensure that ozone is produced effectively and the system is stable.

• The system of the electrolytic ozone generation has multiple functional units that complement each other to produce ozone at a stable and efficient level. The central part of the system is the electrochemical cell that has a membrane electrode assembly where the anode is a boron-doped diamond that is attached to a proton exchange membrane. It has the advantage of efficiently transferring charges and transporting protons and at the same time maintaining physical separation between the anodic and cathodic reaction environments thus avoiding mixing of reaction products.

• The electricity needed in the process is provided by a special direct-current source that gives constant voltage output and a high current density with low electrical variation. The electrical parameters need to be controlled so that they will prefer the generation of ozone at the anode and increase the overall energy efficiency in electrolysis.

• The system is designed to maintain high-purity water in a continuous circulation loop to guarantee a steady electrochemical performance, which is usually characterized with a resistivity that is excessive to 18 M^−1^ cm. Ultrapure water prevents the interference of ions dissolved in the water and minimizes the chances of electrode fouling, as well as degradation of the membrane. A low-temperature range of capabilities of the operating temperature is regulated with the help of an external cooling unit that keeps the temperature in a controlled low-temperature range, usually between 5 and 20 °C. This temperature regulation is necessary to minimize the ozone breakdown and ensure the steady ozone concentration of the aqueous phase.^110^

• Hydrogen gas produced at the cathode is controlled using another treatment unit that is safe to handle *e.g.* controlled collection or catalytic conversion to avoid build-up and increase the probability of explosion. In operation, constantly, deionized water is added to the anode chamber and it is electrochemically oxidized to produce dissolved ozone. At the same time protons flow through the proton exchange membrane to the cathode and are reduced to hydrogen gas.

The electrolytic ozone generator is a system that requires a constant monitoring and feedback control of the most important variables such as water purity, applied voltage, and system temperature to operate steadily. The changes in these parameters may negatively impact on the efficiency of the generation of ozone and the stability of its operation. Electrolytic ozone systems have a number of challenges inherent to their safety and durability aspects. Hydrogen evolution poses possible risks of explosions, whereas the dependence on the high purity of water is susceptible to contamination and system failure. Moreover, long-term performance can be compromised due to degradation of the membranes, deactivation of electrodes, and unstable performance of power supply, which can cause variability in ozone generation and higher maintenance requirements.^111^

#### 3.3.2. Electrode surface design for enhanced electrochemical ozonation

The development of electrochemical techniques of dissolved ozone generation has been progressing very fast within the recent years, with the majority of studies being focused on the enhancement of the anode performance. Specifically, the choice and the adaption of the electrode materials have played a primary role in the increase of the ozone production rates, enhancement of faradaic efficiency, and cause stability in the operation of the technology over longer periods. According to the literature on the topic, three material systems have been predominant in the current research and they include boron-doped diamond (BDD), lead dioxide (PbO_2_), and doped tin oxide (SnO_2_) electrodes. BDD electrodes are frequently listed among them due to their atypically large anodic stability window and high corrosion resistance under severe electrochemical conditions. More recent research has revealed that by tuning crystal morphology and adding controlled heteroatom dopants, BDD electrodes can be used to achieve large current densities without the sharp reduction in ozone efficiency, and therefore can be used in the high-loads electrolysis systems.^112^ Though these have these benefits, the multifaceted complexity of the fabrication and the cost of materials still makes them impractical in large scale or cost sensitive application. Despite their excellent electrochemical stability and high ozone generation efficiency, boron-doped diamond (BDD) electrodes face several practical challenges that limit their widespread industrial adoption. The fabrication of BDD electrodes typically requires sophisticated chemical vapor deposition (CVD) processes and high-purity diamond substrates, which significantly increase production costs compared with conventional metal oxide electrodes. These manufacturing requirements also restrict the scalability of BDD electrodes for large-area industrial electrochemical reactors. Furthermore, although BDD electrodes demonstrate strong resistance to corrosion and oxidation, long-term operational studies indicate that gradual changes in surface structure and boron distribution may influence electrochemical performance during extended operation. Consequently, the overall life-cycle cost of BDD-based ozone generation systems remains relatively high, particularly when considering electrode replacement, system maintenance, and energy consumption under high current densities. For these reasons, current research efforts increasingly focus on improving electrode durability, reducing fabrication costs, and developing hybrid electrode materials that combine the catalytic advantages of BDD with the economic feasibility required for large-scale water treatment applications.^121,125^

Alternatives The lower cost PbO_2_ electrodes have been investigated and have been optimized extensively in terms of structure to improve electrochemical activity. Nanostructured and three-dimensional PbO_2_ architectures enhance the surface area and the mass transfer in the vicinity of the electrode–electrolyte interface, leading to better ozone generation and less energy use.^113^ Previous literature found that in standard membrane electrode assemblies, performance was not stable when used intermittently, but ganged PbO_2_ designs have shown to be more stable under the same conditions.^114^ Simultaneously, β-PbO_2_ nanoparticle production has improved by lowering the prices of raw materials without compromising the power to produce ozone.^115^ However, the issue of electrode degradation, as well as the environmental health risk posed by the lead leaching, continue to be major barriers to long-term implementation.

From a regulatory perspective, the potential release of lead species from PbO_2_ electrodes raises important environmental and public health concerns. Lead is classified as a toxic heavy metal with significant bioaccumulation potential, and its concentration in drinking water is strictly regulated in most jurisdictions. For example, the World Health Organization (WHO) recommends a guideline value of 10 µg L^−1^ for lead in drinking water, while the United States Environmental Protection Agency (US EPA) specifies an action level of 15 µg L^−1^ under the Lead and Copper Rule. Even trace levels of lead leaching from electrochemical electrodes may therefore present regulatory compliance challenges in water treatment applications. Consequently, systems employing PbO_2_ electrodes must incorporate appropriate engineering controls such as stable electrode coatings, corrosion-resistant substrates, and continuous monitoring of dissolved metal concentrations during operation. These regulatory considerations have stimulated increased research into alternative electrode materials with lower environmental risk, including boron-doped diamond and doped tin oxide electrodes, which offer improved chemical stability and reduced potential for toxic metal release in long-term electrochemical ozonation systems.^113^

The current most exciting field of electrochemical ozone generation activity is the use of doped SnO_2_ electrodes. Essentially, a broad spectrum of modification approaches such as elemental doping, co-doping, defect engineering, heterojunction formation and carbon based composites has been utilized to prefer ozone formation to the competing oxygen evolution reaction. The Sb-doped SnO_2_ electrodes have been observed to be economical, however, the problem of dopant instability and gradual leakage still remains a limitation to the life of the electrode. According to recent research, dopant loss can be suppressed by such structures and multi-element doping, and, at the same time, the selectivity of the ozone process and operational stability are enhanced.^116^ Additional performance improvements have been associated with the alteration of intrinsic catalytic pathways instead of surface morphology alone, especially in electrodes of various dopant species.^117^ Despite this, lack of selectivity and sustainability during persistent operation is still a major commercialization challenge. Despite substantial progress in electrode material development, reported performance improvements in electrochemical ozone generation vary considerably across different studies. For example, boron-doped diamond electrodes are widely reported to exhibit superior corrosion resistance and high anodic stability; however, their ozone generation efficiency can vary significantly depending on crystal structure, dopant concentration, and electrode surface morphology.^112^ Similarly, although PbO_2_-based electrodes have demonstrated promising ozone production performance in several studies, concerns remain regarding long-term electrode stability and potential environmental risks associated with lead leaching.^113,114^

Furthermore, conflicting results have been reported regarding the effectiveness of different doped SnO_2_ electrode systems. While some investigations report significant improvements in faradaic efficiency through multi-element doping strategies, other studies indicate that dopant instability and gradual loss of catalytic activity can limit long-term operational performance.^116,117^ These discrepancies highlight the need for systematic durability testing and standardized performance evaluation to better understand the mechanisms governing electrochemical ozone generation and to identify electrode materials capable of stable long-term operation.

In addition to the material composition, the electrode substrate and the reactor design in general has a powerful impact on the production of dissolved ozone. Support of titanium contains mechanical strength and is resistant to corrosion, whereas the use of carbon nanotubes has been demonstrated to increase electrical conductivity and mass transport to increase the levels of ozone in treated water.^118,119^ On the system scale, continuous-flow reactors as well as stacked electrolytic cell designs and structures have been designed to enhance heat dissipation and transport effectiveness, thus enhancing overall ozone productivity. Moreover, ozonized water produced by electrolytes has been used to inactivate microorganisms with significant success in proving to be a viable disinfection system in practical uses.^120^

Conclusively, despite the fact that much has been done in terms of developing electrodes to use in electrochemical dissolved ozone production, a credible long-term working performance data have not been established. Lack of systematic durability studies and ozone stability testing remains a barrier to the adoption of these technologies into the laboratory research to the fullness of the water treatment application. The main results of recent representative studies are outlined in Table 5.

**Table 5:** Ozone yield characteristics under electrochemical operating conditions

S. no.	Year	Research (short)	Current density, *j* (mA cm^−2^)	Reported faradaic/coulombic efficiency for O_3_ (%)	Ref.
1	2016 (review)	The review summarizes aqueous-cell studies and typical current efficiencies in neutral systems	Various (literature summary; examples at tens–hundreds mA cm^−2^)	∼5–12% (typical for many aqueous/neutral electrolysis reports summarized)	121
2	2021 (Ni–Sb–SnO_2_ family, literature)	Reports markedly higher O_3_ selectivity for Ni–Sb–SnO_2_ electrodes compared with earlier oxides (∼30% in early studies)	Often ∼50–150 mA cm^−2^ in practical tests (varies by setup)	∼30% reported in the Zhang *et al.*^120^ example; some recent reports for optimized doped SnO_2_ approach higher values	122
3	2023 (da Silva *et al.*, E3L PEM cell)	3-D-printed PEM concept cell (E3L) for gaseous ozone production; tested current densities and compared energy & coulombic efficiency	50, 100, 150, 200 mA cm^−2^ (authors highlight 150 mA cm^−2^ as most efficient)	≈50% coulombic efficiency at 150 mA cm^−2^ (authors report up to ≈50% and energy efficiency ≈9–10 mg O_3_ per Wh under best conditions)	123
4	2024 (zero-gap BDD/sp^2^ tuning preprint)	In zero-gap BDD cells, carbon content and electrode structure govern current efficiency	Reported tests across tens to several hundreds mA cm^−2^ (cell dependent)	Peak current efficiency increases with optimized sp^2^ content; reported maxima vary by electrode	124
5	2025 (*RSC Review*/Liu *et al.*, 2025)	Recent review on electrochemical ozone production summarizing state-of-the-art electrocatalysts (including Pb-oxide, Ni/Sb–SnO_2_ and BDD systems)	Examples highlighted around 100 mA cm^−2^ (common benchmarking point) and higher	Reports up to ∼50% FE in optimized MEA/flow cells at ∼100 mA cm^−2^	125
6	2026 (*AIChE*/application paper electrochemical ozone for synthesis)	Recent application-focused studies demonstrate stable ozone generation at high current densities, with 500 mA cm^−2^ chosen for stability tests	∼500 mA cm^−2^ (working point chosen for reported electrochemical testing)	Authors emphasize stable ozone production at high current density, with catalyst- and cell-dependent FE.	126

#### 3.3.3. System-level advantages and drawbacks of electrolytic ozone generators

The emerging interest in electrolytic ozone generation as an alternative method of ozone production has had some specific benefits, but still has some critical technical limitations. A direct production of ozone in aqueous solution in relatively high concentrations with the help of a single electrolyte, without air drying, oxygen enrichment, or sophisticated gas pre-treatment units is among the key strengths. This simplified design makes the technology, specifically applicable in applications where high purity and control are needed, including, but not limited to, medical sterilization, food processing, and precision manufacturing. However, there are still a number of obstacles that limit its further use in industry. The higher cost of the specialized electrode material and continuous performance under highly oxidative environments are major factors that bring up the cost of the system and the degradation of the electrode material, lead to poor service life, respectively. Moreover, electrolytic ozone systems tend to consume more energy than traditional ones, and the long-term operational experience in industrial conditions is not sufficient.^127^

When the results from recent studies are considered collectively, it becomes evident that the future development of ozone generation technologies will depend on integrated system optimization rather than improvements in a single technological component. For example, while DBD systems provide superior scalability and operational maturity for industrial applications, electrochemical systems offer advantages in terms of compact design and the ability to generate high-purity dissolved ozone. Recent research increasingly emphasizes improvements in electrode materials, advanced power electronics, and intelligent process control to enhance ozone yield while minimizing energy consumption and operational instability.^112,116^ These developments suggest that hybrid optimization strategies combining advances in materials science, reactor engineering, and system control may significantly improve the efficiency and sustainability of ozone generation technologies in the future.

These restrictions spell out the economic viability of the technology at large scale. Currently, electrolytic ozone production is best suited to small to medium value, high-performance and cleanliness oriented applications where cost is not the paramount factor. The future development will be based on the electrode durability, efficiency in energy usage and coming up with a cheaper production process. Though this technology offers a clean and efficient substitute to chemical disinfectants, there is no possibility that in the near future it will be used to substitute the well-established and economical dielectric barrier discharge (DBD) ozone systems used in large-scale industrial practices. It will be highly reliant on the further developments in materials science and electrochemical engineering to become a viable business in the long run.^128^

### 3.4. Life-cycle economic analysis of ozone generation technologies

**Fig. 6:**
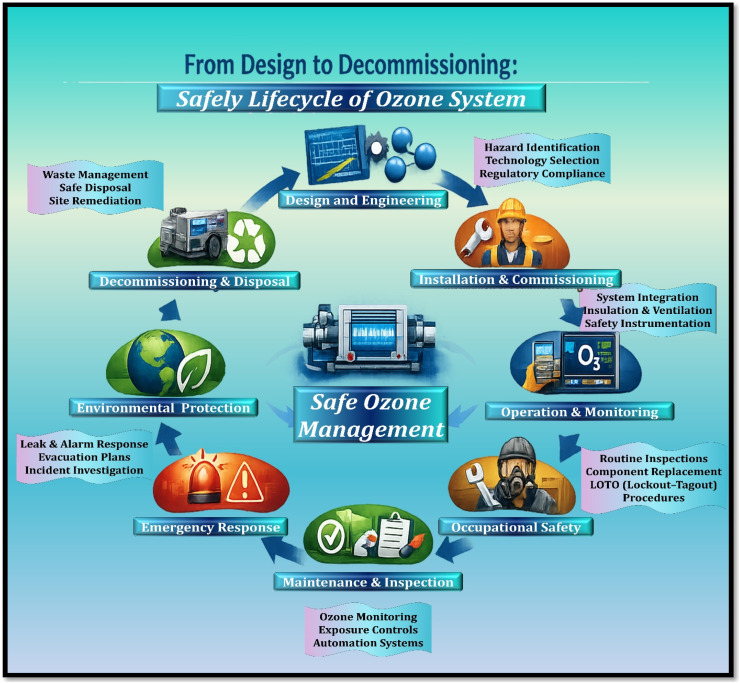
From design to decommissioning: a comprehensive safety lifecycle for ozone technologies.

Engineering-wise, the cost-effectiveness of ozone generation systems cannot be determined only on the premise of the initial capital investment. The demand of operational electricity, the intensity of maintenance, long life of equipment and the indirect expenses on safety management and adherence to regulations have a significant impact on the overall economic performance. The computation of these interacting factors requires a lifecycle cost (LCC) approach to be used to over comparatively evaluate UV, dielectric barrier discharge (DBD), and electrolytic ozone generating technologies, as summarized in Table 6. Simultaneously, safety lifecycle of the ozone system including its design and operation is depicted in Fig. 6.

**Table 6:** Comparative life cycle cost modelling of corona discharge, UV, and electrolytic ozone generators^129^

S. no.	Item	UV ozone generator	DBD ozone generator	Electrolytic ozone generator
1	Capital cost	Low	Medium–high	Medium–high
2	Operating cost	Medium (lamp replacement, electricity)	Medium (electricity dominant)	Medium (electricity, water conditioning)
3	Energy efficiency	Low	High	Medium–high
4	Maintenance	UV lamp replacement, cleaning	Electrode/dielectric and power unit maintenance	Cell/membrane and water system maintenance
5	Typical lifespan	Low–medium	High	Medium
6	Major LCC driver	Lamp replacement + low ozone yield	Electricity + reactor maintenance	Cell durability + water quality
7	Ozone output capacity	Low	Medium–very high	Medium–high
8	Main safety risks	UV exposure, ozone leakage	High voltage, ozone leakage	Ozone leakage, hydrogen generation
9	Safety controls	UV shielding, interlocks, ozone monitors	Electrical insulation, ozone monitors	Hydrogen ventilation, ozone monitors
10	Regulatory focus	Ozone exposure limits, UV safety	Ozone exposure, electrical safety	Ozone exposure, hydrogen safety
11	Overall LCC ranking*	Moderate (small scale)	Low (medium–large scale)	Moderate (small–medium scale)

The benefits of UV ozone generators include the fact that they are easy to build, cost-effective and require little capital.^130^ They use low- or medium-pressure UV lamps in a simple reactor setup which does not require high-voltage parts.^131^ Such simplicity allows them to be used in laboratory-scale, decentralized or intermittent applications. Nevertheless, they have a low efficiency in their production of the ozone, and therefore have high energy requirement per unit ozone produced. Moreover, the aging process of UV lamps and regular change is a factor that decreases the long-term economic reliability.^132^ Although the costs associated with safety, including UV shielding and disposal of mercury wastes are low at small scales, they grow when there is continuous operation, which limits their competitiveness at large-scale systems.^133^

The most established type of ozone generator is the dielectric barrier discharge (DBD) ozone generators which are used in industry. These discharge reactors, cooling systems, and safety systems are more expensive in terms of high-voltage power supplies, but the expenses are fairly controlled with the help of unified and scalable designs. DBD systems have high ozone production with relatively low power usage which makes it have the lowest life-cycle cost, especially when used continuously and in large scale. Maintenance is a foreseeable cost, and the cost of compliance related to high-voltage operation and ozone containment is highly standardized, and its cost remains relatively small. In this regard, therefore, DBD technology offers the most balanced performance in cost, efficiency and scaling.^134^

There is less economic attractiveness in the electrolytic ozone generators. Advanced electrodes, ion-exchange membranes, and the requirement of high-purity water systems are the reason why the cost of capital is high.^135^ High electricity cost, material destruction and other safety precautions needed to manage the hydrogen by-products increase operational costs.^136^ These systems have the ability to produce high concentrations of dissolved ozone in water but this does not justify the overall operating and maintenance costs.^137^ Consequently, only a few or high-value processes utilize electrolytic generators. In general, it is possible to note that UV generators are the most appropriate in small-scale or intermittent applications, DBD generators have the most economic and scalable solution in industrial applications, and electrolytic systems are limited to the niche applications with their particular benefits and higher costs.

Although individual ozone generation technologies have been widely investigated, direct comparative evaluations remain relatively limited. UV-based ozone generation systems are generally characterized by simple reactor configuration and low capital investment; however, their ozone production rate and energy efficiency are significantly lower compared with plasma-based technologies. Consequently, UV systems are mainly suitable for small-scale applications where simplicity and operational safety are prioritized over production capacity. In contrast, dielectric barrier discharge (DBD) systems currently represent the most mature industrial technology for ozone generation. Their ability to produce high ozone concentrations with comparatively favorable energy yield makes them suitable for continuous large-scale operations such as municipal water treatment and industrial oxidation processes. Nevertheless, DBD systems require complex high-voltage power supplies and efficient thermal management to maintain stable plasma conditions, which increases system complexity. Electrochemical ozone generation provides a fundamentally different approach by producing ozone directly in aqueous media. This feature enables high-purity dissolved ozone production without gas transfer limitations, making the technology particularly attractive for medical sterilization and high-value water treatment applications. However, electrode degradation, high material costs, and relatively high energy consumption remain major barriers to widespread industrial adoption.

Overall, current evidence suggests that no single ozone generation technology universally outperforms others across all performance metrics. Instead, the optimal technology depends strongly on application requirements, including ozone concentration demand, system scale, operational cost constraints, and safety considerations. Despite the considerable progress in ozone generation technologies, several important knowledge gaps and unresolved challenges remain. One major limitation is the absence of standardized performance evaluation frameworks across different ozone generation methods. Many studies report parameters such as ozone concentration, energy yield, or specific energy consumption under different experimental conditions, which makes direct comparison between technologies difficult.^43,47^ Conflicting findings have also been reported regarding the influence of operational parameters on ozone generation efficiency. For instance, some studies suggest that moderate humidity levels may stabilize plasma discharge and improve ozone formation in dielectric barrier discharge reactors, whereas other studies report that increased moisture accelerates ozone decomposition and decreases overall ozone yield.^88,89^ These inconsistencies highlight the need for systematic experimental investigations under controlled conditions.

In addition, the long-term operational stability of emerging ozone generation technologies remains insufficiently understood. Electrochemical ozone generation systems often face challenges related to electrode degradation, membrane fouling, and declining current efficiency during prolonged operation.^112,127^ Similarly, dielectric barrier discharge generators may experience dielectric aging, electrode erosion, and thermal instability under continuous high-voltage operation.^86^ Furthermore, large-scale industrial implementation of advanced ozone technologies remains constrained by unresolved issues related to energy consumption, reactor scalability, and integration with existing treatment systems. Although recent studies report significant improvements in plasma reactor design, electrode materials, and power electronics, further research is required to develop energy-efficient, durable, and economically viable ozone generation systems suitable for continuous industrial applications.^134^ To provide a clearer comparison of the technological characteristics of different ozone generation systems, the key performance indicators and operational considerations of major ozone generation technologies are summarized in Table 7.

**Table 7:** Quantitative comparison of major ozone generation technologies

Technology	Typical ozone concentration	Specific energy consumption (SEC)	Scalability	Major safety considerations	Key references
UV photochemical ozone generation	0.01–0.2 wt% in gas phase	80–150 kWh per kg O_3_	Low–moderate	UV radiation exposure, ozone leakage, lamp breakage	47 and 106
Dielectric barrier discharge (DBD)	1–10 wt% (oxygen feed systems)	8–20 kWh per kg O_3_	High (industrial scale)	High-voltage electrical hazards, ozone leakage, NO_*x*_ formation in air-fed systems	47, 106 and 125
Electrochemical ozone generation	100–300 mg L^−1^ dissolved ozone	20–60 kWh per kg O_3_	Low–moderate	Hydrogen evolution, electrode degradation, membrane fouling	121, 125 and 127

## 4. Ozone applications in environmental and industrial processes

**Fig. 7:**
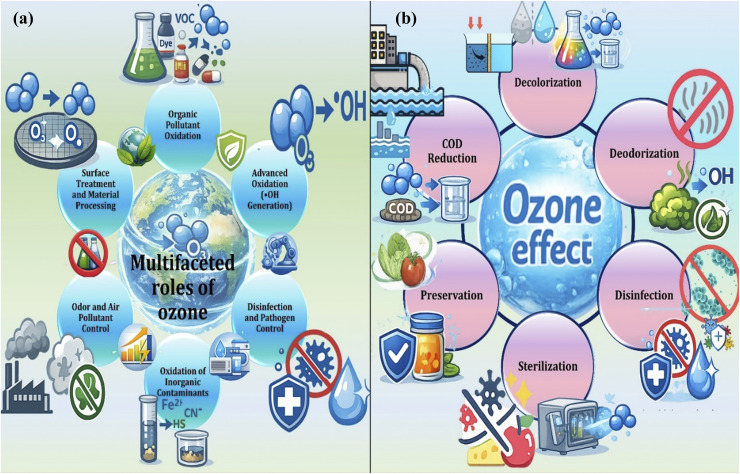
(a) Multifaceted roles of ozone in environmental remediation and industrial processes, emphasizing its strong oxidative capacity, broad-spectrum reactivity toward organic and inorganic contaminants, and versatility across air, water, and surface treatment applications. (b) Fundamental mechanistic understanding of ozone-driven water treatment, illustrating direct molecular ozone reactions and indirect pathways mediated by reactive oxygen species, which together govern contaminant degradation efficiency and selectivity. Reproduced from *Atmospheric Chemistry and Physics*,^139^ © 2020, distributed under the Creative Commons Attribution 4.0 License (CC BY 4.0).

Ozone is a strong oxidizer that is deemed safe to the environment since it disintegrates rapidly to oxygen without leaving any lasting and harmful by-products.^138^ Such a combination of high reactivity and environmental friendliness have helped its increased application in fields like environmental protection, in the health of the people, and food industry. Ozone has a high degree of flexibility because it can work in a broad spectrum of applicable conditions as shown in Fig. 7a. This review will address four primary fields of use of ozone in water treatment, air purification, food preservation, and medical uses and both discusses the mechanisms underlying such uses and the most recent achievements of ozone-based technologies.^140^

### 4.1. Environmental water treatment approaches

The notion of using ozone as a tool of sophisticated and sustainable water treatment has become very popular. Its use increases the efficiency of purification, in addition to adhering to the increased need of disinfection techniques with environmental friendliness.^141^ Ozone can quickly neutralize pathogenic microorganisms, dissolve the recalcitrant organic contaminants and enhance water appearance and odor properties are demonstrated in Fig. 7b because of its high oxidation potential. Ozone does not linger in the treated water, in comparison to chlorine-based disinfectants, but rather breaks down into oxygen, thus eliminating the possibility of creating disinfecting by-products (DBPs) that are hazardous and carcinogenic.^142^ Most lately, the advanced oxidation processes (AOPs) using ozone have begun to be implemented more frequently as part of a treatment train in drinking water production, wastewater reuse, and control of industrial effluents. These developments have made ozone become a fundamental technology in contemporary water treatment practice. Table 8 summarizes its applications under the different operating conditions.

**Table 8:** Role of ozone in the removal of refractory and emerging contaminants

S. no.	Application scenario	Target pollutant	Ozone-based process	Removal effect (%)	Ref.
1	Drinking water disinfection	Bacteria (*E. coli*, *Vibrio cholerae*)	Direct ozonation (O_3_ molecular oxidation)	99.9	143
2	Drinking water disinfection	Viruses (enteroviruses, norovirus)	Direct ozonation	99.9	144
3	Protozoa control	Giardia, cryptosporidium	Ozonation (cell wall oxidation)	99.9	145
4	Taste and odor control	Geosmin, 2-MIB	Direct ozonation	80–95	34
5	Color removal in surface water	Humic substances	Ozonation	70–90	41
6	Natural organic matter (NOM) reduction	Fulvic & humic acids	Ozonation + biofiltration	40–70	47
7	Disinfection by-product precursor control	THM & HAA precursors	O_3_ + biological activated carbon (BAC)	50–80	77
8	Iron and manganese removal	Fe^2+^, Mn^2+^	Ozonation followed by filtration	90–99	79
9	Industrial wastewater treatment	Phenols	O_3_ oxidation	70–95	85
10	Textile wastewater	Reactive & azo dyes	Ozonation/O_3_–H_2_O_2_ (peroxone)	80–99	89
11	Pharmaceutical wastewater	Antibiotics, analgesics	O_3_/O_3_-UV	70–98	105
12	Pesticide removal	Atrazine, chlorpyrifos	O_3_–H_2_O_2_ (AOP)	60–95	109
13	Endocrine disrupting compounds	Bisphenol-A, hormones	O_3_/O_3_-UV	70–99	111
14	Cyanotoxin control	Microcystin-LR	Ozonation	90–99	76
	Algal control	Algae cells	Pre-ozonation	60–90	139
15	Municipal wastewater tertiary treatment	COD	O_3_/O_3_–H_2_O_2_	30–60	122

#### 4.1.1. Potable water treatment and management

**Fig. 8:**
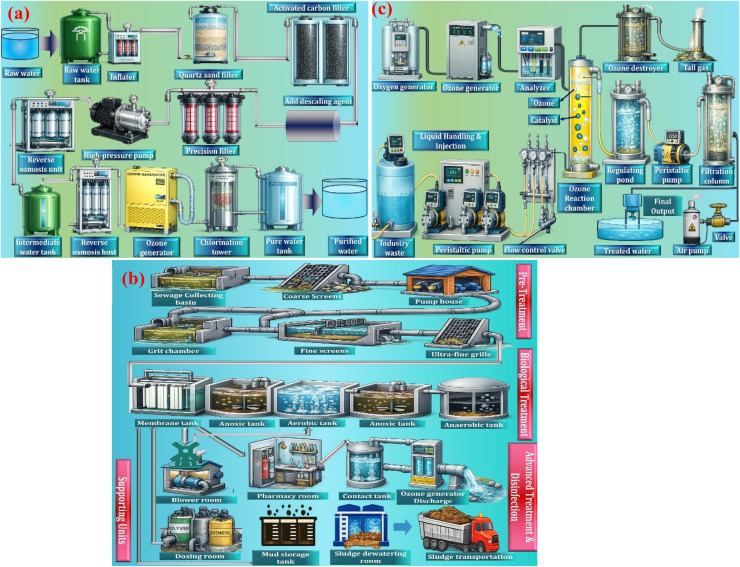
Comprehensive applications of ozone in modern water treatment systems: (a) the process flow for ozone treatment for drinking water; (b) the urban sewage treatment (c) the process flow for Industrial waste water treatment. Reproduced from Elsevier,^146^ © 2022, under a Creative Commons license.

Safety of drinking water is a basic need towards the health and protection of the environment. Ozone has been extensively used in advanced treatment systems due to its high oxidative capacity and hence can be used to inactivate recalcitrant pathogens and decompose trace-level organic contaminants are illustrated in Fig. 8a. As soon as ozone is introduced into water, it breaks down very quickly, with hydroxyl radicals (˙OH) formed which results in an extremely reactive oxidative atmosphere that is able to neutralize microorganisms very fast.^147^

The ozone disinfection does not have a single lethal mechanism but a number of pathways. It combines with key cellular components, such as enzymes, proteins, and nucleic acids, resulting in dysfunction of the metabolism and reproductive incapacity. Significantly, it is not always accompanied by visible membrane rupture and intracellular substance leakage process. It has been demonstrated in the previous research that cells can be found structural intact, but they can turn nonviable after being exposed to ozone. This is because of the broad and simultaneous oxidative action; ozone is able to inactivate a wide range of microorganisms, including bacteria, viruses, and chlorine resistant organisms, in just few to several minutes.

Ozonation has been considered as a safe advanced treatment process of drinking water due to its capacity to be very powerful in oxidation and disinfection. Ozone is able to inactivate resistant microorganisms including bacterial and fungal spores much faster than chlorine-based methods. Experimental investigations have shown that comparatively low levels of ozone could result in high levels of microbial inactivation, though there is a high dependence on the quality of water and the composition of the matrix. Ozonation is commonly used together with a biological activated carbon, or other post-treatment processes, in order to enhance stability in operations, and guarantee consistent control of pathogens.^148^

Besides disinfection, ozone is also very effective in eliminating organic contaminants including taste and odor causing compounds, natural organic substances and emergent pollutants. Ozone reactions destroy chromophoric structures and functional groups, and decrease odor intensity, and converting large organic molecules into smaller, more biodegradable fractions. Electrochemical enhanced ozonation processes additionally enhance the removal of the contaminant and reduce bromates especially in waters containing bromide hence balancing the treatment performance and concentrations of by-products. Ozonation also minimizes the formation potential of disinfection by-products that may form on carbon base during the subsequent chlorination process in degrading high-molecular-weight organic matter. Nevertheless, when bromide is present, ozone can produce bromate and low-molecular-weight oxygen by-products, which can support the growth of microbes. Such risks can be controlled well by using downstream treatments, which may include BAC filtration, addition of phosphate or activated carbon systems. In general, ozonation is an effective and flexible treatment method, the effective implementation of which cannot be done without the careful combination and optimization depending on the local water quality and operational parameters.^149,150^

#### 4.1.2. Municipal wastewater treatment systems

The source of municipal wastewater is a mixture of domestic operations, industrial effluents, and storm water flowing into sewer systems during rainy seasons.^151^ Released inadequately treated, this wastewater may serve as a carrier of infectious diseases, and alter aquatic ecosystems and create long-term economic and social costs. These issues render the application of the high-tech wastewater treatment the necessity to keep the health of people safe as well as ensure the sustainability of the water resources.

Ozone-based advanced oxidation processes are typical of modern-day urban treatment facilities employed in their tertiary treatment phase to enhance the quality of effluents further as illustrated in the Fig. 8b schematically. Ozone is a potent oxidizing agent and when combined with any other process, it facilitates the production of high reactive radical species. This allows efficient inactivation of recalcitrant microorganisms and conversion of refractory organic pollutants that are hard to get rid of by conventional methods. Simultaneously, ozone treatment enhances aesthetic quality of water by eliminating colour, odour and taste causing substances. The combination of these oxidation processes allows for further pollutant degradation and mineralization, which contributes to the reuse of the quality of effluent and enhance the application of the ozone-based technology in satisfying future stricter requirements of municipal wastewater treatment.

Advanced oxidation processes (AOPs) that involve the use of both ozone have been found to be effective in enhancing removal of recalcitrant micropollutants. Combination of ozone and ultrasound, photocatalysis, hydrogen peroxide, or cavitation has markedly improved efficiency of oxidation through a rise in production of reactive oxygen species. Pilot- and laboratory-scale experiments show invariably higher degradation rates and removal efficiencies of pharmaceuticals including ibuprofen, carbamazepine, and gemfibrozil than of standalone ozonation. Constant photocatalytic ozonation has also shown itself to be effective at removing micro-pollutants, antibiotic resistance genes (ARGs) and also estrogenic activity, usually to levels where they are no longer detectable. All in all, hybrid ozone systems are superior to single ozone processes, but the majority of data has only been tested on smaller scales, which has to be properly validated at full-scale.^152^

Ozone can also be used to disinfect wastewater effectively because it has a high oxidative capability against the cell structure of microorganisms, viral capsids, and nucleic acids. It rapidly inactivates bacteria and viruses and breaks resistance genes sequences, which helps to reduce the dissemination of ARGs. Research demonstrates that ozone easily inactivates microorganisms as opposed to cell-associated ARGs, and the degree of removal is highly reliant on dose applied.^153^ The moderate levels of ozone can be used with a great success in the case of resistant bacteria and viruses, but higher doses are commonly needed to break down ARG. Ozone is better than the traditional disinfectants in that it has excellent microbial control and reduced chances of causing secondary resistance. Although ozonation has some advantages, there are some potentially harmful by-products which can be produced by ozonation including bromate and brominated organics especially in water with a high concentration of bromide. Activated carbon post-treatment has been demonstrated to be an effective method of ecotoxicity reduction in water and overall water quality compared to the biological filtration and membrane-based polishing steps. Although more sophisticated methods like oxidation titration can be used to maximize the usage of ozone and minimize the generation of by-products, their use at a large scale is uncertain.^154^

Sludge treatment also involves the use of ozone which alters the structure of flocs, cell lysis and dewatering efficiency. The ultrasound-assisted, catalyzed and flotation-based ozonation methods have also exhibited enhancement in sludge thickening, pathogen elimination, and reduction in toxicity. Nonetheless, the complexity of processes and their scalability are major issues. Considering the economic and operational point of view, the ozone-based upgrades provide a solution to the advanced wastewater treatment in a cost-effective manner. Competitive costs of treatment and high level of micro pollutant removal, especially in combination with activated carbon or membrane systems, are demonstrated by full-scale tests. However, the performance is greatly location-dependent and it needs site-specific optimization of the process. On the whole, the ozone-based technologies are a flexible and effective solution in the treatment of advanced municipal wastewater that allows to control simultaneously micro pollutants, pathogens, and antibiotic resistance.^155^

#### 4.1.3. Treatment technologies for industrial wastewater

Industrial wastewater is generated by many manufacturing and processing processes and may include a heterogeneous blend of contaminants in high levels, such as those that are poisonous or toxicant to life.^156^ These properties often undermine the effectiveness of the traditional biological treatment procedures, and high-level treatment plans are required. As a consequence of this, ozone has widely been used in industrial wastewater treatment because of its high oxidizing ability, which allows the effective breaking down of the stubborn organic pollutants, elimination of color, and inactivation of microorganisms are depicted in Fig. 8c.^157^ Real life applications of ozonation methods are normally provided as pre-treatment or advanced treatment method to increase biodegradability and downstream operation, though it is necessary to ensure strict control on operation to reduce the generation of undesirable by-products like bromate.

Ozone-electrochemical hybrid methods have turned out to be very effective in treatment of industrial wastewater with large amount of persistent pollutants. In such systems, electrochemical reactions are produced inside the reactor, which synergistically reacts with ozone to boost the performance of oxidation overcoming major weaknesses of traditional ozonation including high selectivity and high energy cost. As an example, used ozonation and electrocoagulation on the steel industry effluent, attaining a 99.8 percent cyanide, 94.7 percent COD, and 95 percent BOD and was more cost-effective than other hybrid methods. Likewise, have also found that ozonation aided with electrocoagulation allowed the removal of color (more than 95 per cent in 18 min) in textile wastewater, less toxicity, and minimized ozone use.^158^

Higher levels of integrations also improve the efficiency of treatment. In a three-dimensional electrochemical reactor that integrates electrolysis, ozonation, and activated carbon particles as electrodes was developed, which improved the removal of TOC (95.58%) and degraded nitrobenzene (92.30) because of the enhanced generation of hydroxyl radicals.^159^ Adding of ozone to hydrogen peroxide and Fenton reagents or to persulfates also facilitates the generation of hydroxyl and sulphate radicals and this increases the scope of degradable organic pollutants. To illustrated that sequential ozone-Fenton oxidation was effective in reducing the organic loads and enhancing the biodegradability of diazodinitrophenol wastewater, whereas demonstrated that Fe-based catalytic ozonation was more effective than sole ozone in degrading the hydrophilic organic matter in treated effluent.^160,161^

The catalytic ozonation is also used to enhance the effectiveness of oxidation, catalysts form radicals faster using metal-based or porous catalysts. To determined that cerium-impregnated ZSM-5 was able to remove 86.3 percent of TOC in the treatment of nitrobenzene wastes, whereas asserted that iron filings could dramatically improve the performance of ozonation when applied to coking wastewater, raising the rate of organic removal to more than 80 percent. The use of ozone as a pre-treatment to decompose Macromolecular organics, toxicity, and biodegradability is also common especially in textile and pharmaceutical effluent. The have shown that the pre-treatment of ozone and biological activated carbon significantly enhanced COD, color and organic carbon removal of textile effluent. To demonstrated that with pre-ozonation followed by anaerobic treatment with the membrane bioreactor, 99 per cent of etodolac and about 90 per cent of COD were removed in pharmaceutical wastewater.^162–165^

In general, the use of ozone-based advanced oxidation processes provides good possibilities in industrial wastewater treatment in terms of enhancing the biodegradability, decolourization, and detoxification. Ozone can be used in conjunction with electrochemical or catalytic systems in order to make its use more efficient and economically viable. Nevertheless, it is important to maximize operating conditions and consider by-product formation in order to achieve safe, effective, and sustainable treatment in order to be successfully applied.

### 4.2. Air treatment and control technologies

Air purification technologies have become of more importance to ensure the health of the population, ecosystem protection, and sustainable economic development. They serve to minimise the adverse air pollutants, such as fine particulate matter (PM 2.5) and volatile organic compounds (VOCs) both indoors and outdoors.^166^ The application of ozone as an extremely strong oxidation agent has found widespread implementation in the industrial treatment of waste–gas, the enclosed spaces sterilization, and the in-house air cleaning processes.^167^ It is responsive to pollutants like nitrogen oxides, sulfur compounds and chemically resistant VOCs, and decomposes them to inorganic end products instead of just shifting them across the phases.^168^ Consequently, the secondary pollution is prevented and the necessity to use more chemical agents is minimized. In spite of these benefits, ozone usage in the indoor environment is highly controlled as it may cause health related problems due to over-exposure to respiratory irritation and other related problems. Consequently, in practice, close regulation of the concentration of the ozone, the restriction of human presence, the proper consideration of the exposure time, the sufficient supply of the ventilation is essential to remove the residual ozone immediately.

#### 4.2.1. Industrial emission sources and control

The exhaust gases produced in industries are usually a complex mixture of harmful gases which include sulfur- and chlorine-based substances, nitrogen oxides, odorous species, and highly stable volatile organic compounds (VOCs). Most traditional methods of treatment including adsorption, thermal incineration and biological methods are less effective in treating chemically recalcitrant pollutants. Ozone-assisted oxidation has in these instances provided a good substitute. Ozone may be used either in the upstream of biodegradation of pollutants or attached directly to catalysts to enhance stronger oxidation. This method promotes the breakdown of pollutants, and minimizes the use of more chemical reagents.^169^

**Fig. 9:**
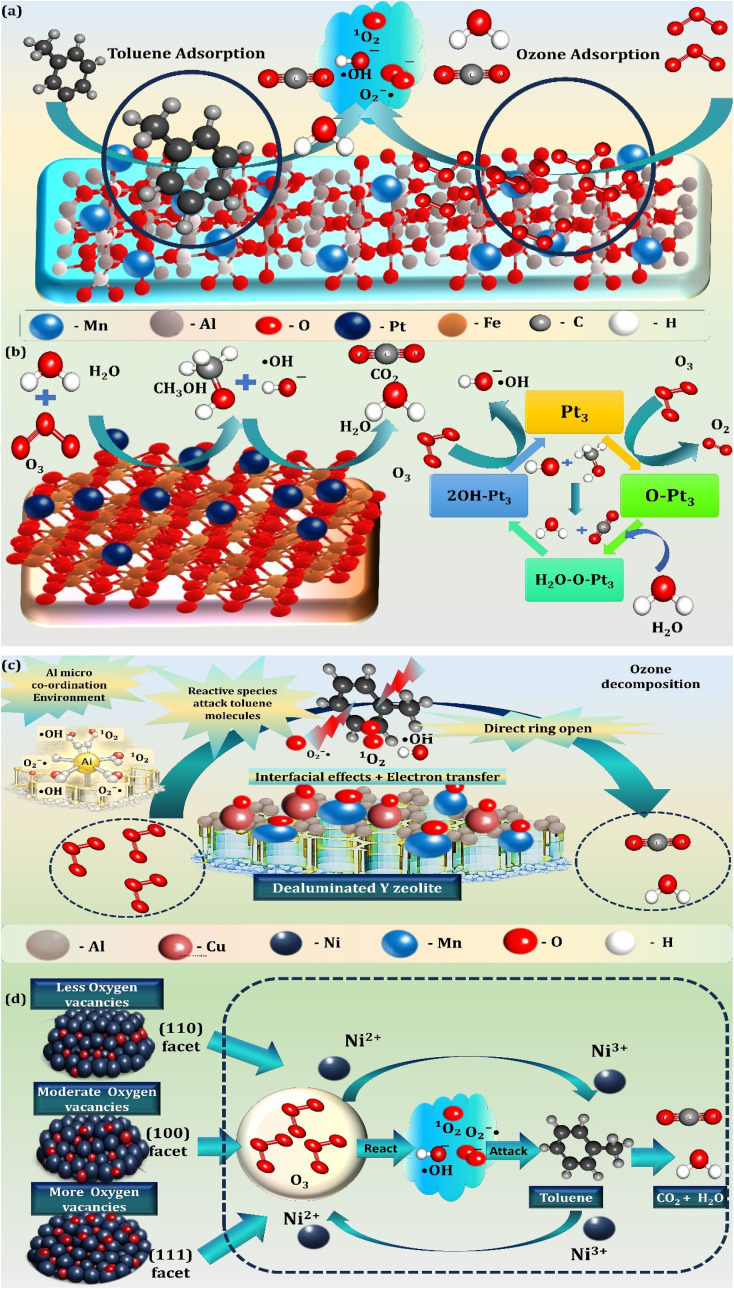
(a) Catalytic ozonolysis of toluene over MnO_*x*_/Al_2_O_3_, highlighting the role of manganese oxide active sites in promoting ozone activation and aromatic ring oxidation.^170^ Reproduced with permission from American Chemical Society (ACS), 2023.^170^ Copyright © 2023 American Chemical Society. License number: 6250790832872. (b) Complete mineralization of gaseous methanol *via* room-temperature catalytic ozone oxidation on Pt/FeO_*x*_ catalysts, demonstrating the strong synergistic effect between Pt nanoparticles and iron oxide supports in low-temperature VOC removal.^171^ Reproduced with permission from American Chemical Society (ACS), 2020. Copyright © 2020 American Chemical Society. License number: 6250791024061. (c) Highly efficient low-temperature catalytic ozone oxidation of toluene achieved over Cu–Mn/DY catalysts, where the dual-metal interaction and optimized support structure significantly enhance ozone utilization and oxidation kinetics.^172^ Reproduced with permission from American Chemical Society (ACS), 2022. Copyright © 2022 American Chemical Society. License number: 6250791248960. (d) Crystal plane-dependent catalytic ozonolysis of toluene on monoclinic NiO, revealing that specific exposed facets markedly improve ozone activation and accelerate deep oxidation of aromatic pollutants.^173^ Adapted with permission from American Chemical Society (ACS), 2023. Copyright © 2023 American Chemical Society. License number: 6250791402703.

The low-temperature catalytic oxidation has been a key research area in the purification of the air, especially in the removal of VOCs under the light-operating environment. Fig. 9 demonstrates that toluene is an economy compound because it is stable and often contains in industrial emissions. In the study the MnO_*x*_/Al_2_O_3_ catalyst was shown to entirely remove toluene at room temperature in the presence of ozone with a high rate of 82.3 percent mineralization. These authors attributed this performance to surface hydroxyl groups, which favor the development of reactive oxygen species. In another study obtained complete oxidation of gaseous methanol to CO_2_ and H_2_O with a Pt/FeO_*x*_-400 catalyst at 30 °C, which showed that ozone-catalyst interactions could make an oxidation pathway significantly faster. In an effort to address the challenge of humid operating environment, to came up with a CuMn/DY catalyst that would be able to maintain high activity by regulating the aluminum coordination environment within the Y-type zeolites purporting to have 95% toluene removal efficiency. More recently, The demonstrated that NiO-based catalysts had the potential to achieve near-complete toluene conversion in 120 minutes at 30 °C under a well-defined concentration of ozone, humidity, and the space velocity.

In addition to VOCs, ozone-enhanced catalysis has been used to treat other toxic components of flue gases, especially sulfur- and chlorine-containing gases.^174^ The study has shown that the existence of ozone enhanced chlorobenzene transformation in the presence of Mn/Al_2_O_3_ catalysts and reduced the catalyst deactivation by SO_2_. There has also been development in the treatment of the multi-pollutant gas streams. The cerium–titanium catalytic oxidation and ammonia absorption which allowed the removal of NO in the environment in addition to SO_2_ and at the same time transform the products collected into ammonium fertilizers. The ammonia-based coupled systems with an emphasis on the possibility to attain both emission management and resource recycling.^175^ There has been a growing need to improve system integration to achieve a practice-based exhaust gas treatment. To demonstrated that the interplay of vacuum ultraviolet photolysis and catalytic ozonation increased the VOC degradation and reduced undesirable ozone emission. To designed a hybrid system of plasma and catalyst, which was able to effectively eliminate ketone-type VOCs with complicated operating conditions. To observed through field observations that alkenes and aromatic hydrocarbons were the primary contributors to urban ozone, which implies that special attention should be paid to VOC control. Simultaneously, To showed that bimetallic metal–organic framework materials would be able to eliminate ozone and VOCs at a broad humidity spectrum, suggesting a high possibility of application in practice.^176,177^

In spite of the positive developments, there are a number of challenges. The majority of ozone-based catalytic systems have been tested at laboratory or pilot levels and little data is available on the long-term stability of catalysts and ozone usage efficiency and functionality under the varying industrial conditions. Moreover, the case of unfinished oxidation of intermediates as well as the potential development of secondary pollutants cannot be overlooked. The work of the future thus should be devoted to the understanding of radical driven reaction mechanisms, to the durability tests in realistic exhaust environments, and to creating multifunctional catalysts with the optimal reactor designs to enable a reliable and sustainable industrial use.

#### 4.2.2. Technologies for indoor and ambient water purification

**Fig. 10:**
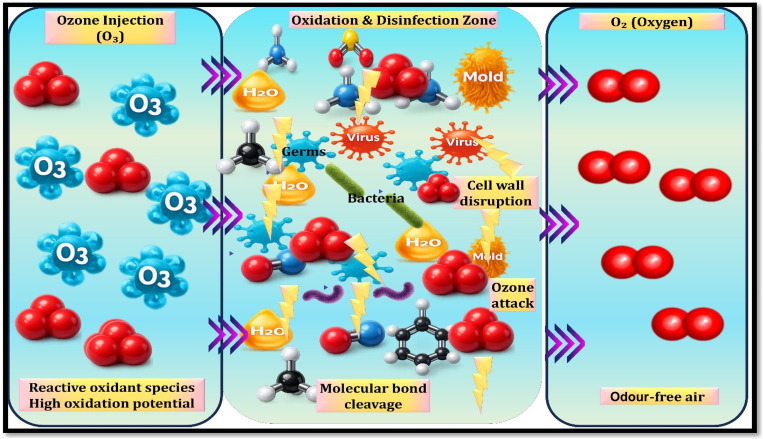
Advanced ozonation strategies for oxidative decomposition of airborne contaminants.

The application of ozone in the indoor setting is primarily in air cleaning where the ozone has been applied to eliminate odours and disinfection due to its high oxidative ability.^178^ It reacts easily with formaldehyde and other offensive substances and has the capability to inactivate a wide variety of microorganisms. The reaction mechanisms are given in Fig. 10.^179^ In spite of such advantages, the use of ozone should be carefully controlled. One should only administer treatment in empty areas and ozone should be well ventilated once it has been treated with ozone to make sure that ozone levels drop to what is considered as safe before the area is reopened.

Recent researches have shown that ozone and plasma technologies are increasingly being used in air sterilization. PEM-based ozone production has been demonstrated to be able to suppress *Klebsiella pneumoniae*, including the down-regulation of antibiotic resistance genes with low ozone production. Combined ozone–manganese dioxide catalyst Hybrid systems with manganese dioxide and ozone have been shown to inactivate bacterial and fungal bio-aerosol with high inactivation efficiency, and at the same time removes residual ozone in treated air. Plasma assisted methods, including dielectric barrier discharge with ion wind or filtration units, have also been found to work in indoor disinfection, providing long-term control of microbes whilst also keeping ozone levels at reasonable levels.^180^

Ozone has also been the considered disinfection agent that is used in general and much attention is paid to safety. Ozonation guidelines have been effective and implemented to disinfect the air and surfaces in empty rooms, especially during emergencies in the population. Nevertheless, relative studies show that low-density ozone does not perform well with respect to air disinfection compared to UVC irradiation, which always has stronger germicidal activity. In order to enhance the ability of application, hybrid systems that combine photocatalysis and photolysis have also been created, allowing the degradation of pollutants and inactivation of microbes at the same time.^181^ Simultaneously, studies in the field of air purification are also placing an increasing focus on combined technologies of volatile organic compounds removal. Catalytic and photocatalytic processes enhanced by the use of ozone have demonstrated better performance on removing formaldehyde and VOCs that are based on ketones, even at low temperature, with little secondary pollution. The development of the optimization of plasma systems has enhanced the efficiency of energy and the use of the ozone.^182^

In general, ozone-based air purification is moving towards multifunctional, hybrid systems, which are capable of disinfection, oxidation, and by-products control. Ozone application will be limited by exposure limits and occupancy requirements despite its good oxidation capacity. Consequently, the use of ozone should be considered as an addition in integrated air treatment systems and cannot be used as a single option to continue and be used indoors.

### 4.3. Food decontamination and preservation applications

The application of ozone in the food industry depends greatly on the location of its introduction to the processing line and the manner in which it is used whether as gaseous or dissolved in water.^183^ The two types of delivery systems are very different in their mass transfer efficiency, reaction pathways, and their impact on food quality parameters.^184^ Ozone is not considered a normal food additive in most of the regulatory systems but as a processing aid.^185^ It can only come into direct contact with the food when conditions of operation and good manufacturing practices are highly adhered to. Ozone decomposes quickly to oxygen, so it does not leave behind as a chemical residue in the final product. Nonetheless, regulations focus on strict control of the conditions of the process, worker safety, and validation processes. Ozone may also cause unwanted oxidative damage to lipids, natural pigments or sensitive nutrients when used incorrectly.^186^ Owing to this fact, before ozone is safely used in food processing processes, the regulatory provisions and prudent evaluation of oxidative hazards must be adhered to.

#### 4.3.1. Gaseous ozone for food decontamination and preservation

Another option that has sparked a lot of interest is gaseous ozone as a residue-free surface disinfectant, environmental hygiene measure, and solid food product storage.^187^ Since ozone is easily dispersible in air, it is able to reach small crevices, uneven surfaces and crannies that are hard to approach with liquid sanitizers. This feature endows it with the ability to decontaminate food-contact surfaces, storage, and bulk commodities.^188^ Meanwhile, the oxidative reactivity of ozone is high and, therefore, the concentration and exposure duration should be carefully controlled because overtreatment can have a detrimental impact on the quality of products.^189^

It is shown through experimental data that gaseous ozone has antimicrobial efficacy in the surface and air cleaning of environmental objects.^190^ It has been used to reduce the number of pathogenic microorganisms on fresh produce, processing equipment, and air of refrigerated storage facilities.^191^ As an illustration, we found that higher doses of microbial were killed quickly with little effects on sensory characteristics, whereas reported the fact that low levels of ozone were enough to disinfect processing surfaces and reduce cross-contamination. Besides that, hybrid plasma-ozone systems have demonstrated higher disinfection capabilities, with microbial reductions exceeding 90 percent in both hospital and food-processing environments.^192^

Ozone gases are also explored as a preservation technique to inhibit microorganisms, as well as delay the degradation of the quality during storage. Research on fruits like grapes and pomegranates has recorded fewer incidences of fungal growth and water loss, retention of color, acidity, and sensory attributes after ozone exposure. Nonetheless, the effectiveness of treatment is diverse and depends on a number of interacting conditions, such as dose of ozone, exposure, humidity relative to the package, and packaging conditions. This is a drawback of meat systems; in their case, have only described significant microbial losses in beef subjected to 310 ppm ozone. All in all, although gaseous ozone has a wide antimicrobial spectrum with no residues, the effectiveness of the technology is extremely sensitive to the conditions of application. This lack of standardization of reporting and especially on the CT values still makes it difficult to compare the studies in any meaningful way, and also makes it difficult to adopt the technique further in industries.^193–195^

#### 4.3.2. Aqueous ozone for food decontamination and quality preservation

The aqueous ozone finds extensive application in the liquid food industry in the inhibition of microbes, modification of ingredients, and sanitation of equipment. Dissolved ozone is better than gaseous because it offers greater distribution and more control over dosage, and thus, it is especially applicable to beverages, tofu and liquid extracts and other high-moisture foods. Research continuously supports that ozonated water is an effective food preservative in terms of inhibiting food violence as well as minimally affecting the nutritional and sensory characteristics of foods. It can also be used in conjunction with organic acids, ultraviolet irradiation, or catalytic systems to further improve its antimicrobial activity without leaving any chemical residues. Besides disinfection, aqueous ozone has found application in the removal of contaminants as well as ingredient modification. It has the capacity to destroy pesticide remains, mycotoxins, and other undesired substances, and enhance functional attributes to cereal and flour systems, which includes water absorption and dough dynamics. Ozone finds applications as well in the cleaning of processing equipment as well as the treatment of waste water that helps in decreasing the chemical oxygen requirement and the environmental sustainability.^196^

In general, aqueous ozone provides a controllable and residue free alternative to traditional chemical food treatment. Nevertheless, its action relies heavily on the concentration of ozone and time of exposure, and the properties of food matrix. The absence of standardised treatment measures and the large number of laboratory-scale studies are the main issues, which means that the protocol needs to be harmonised and tested in industrial environments.

### 4.4. Medical and clinical uses

The high oxidative reactivity of ozone, which allows it to be used in medical disinfection and clinical practice, is mainly due to its high reactivity. Besides the disinfecting power, ozone was found to regulate immune responses and provide tissue healing and regeneration. Ozone is cheap, easy to produce on-site, and easy to apply, and this has made it widely used in environmental sanitation in addition to sterilizing medical devices.^197^ Further, ozone has also been utilized as an aid to therapeutic use in an expanded scope of clinical practice.

#### 4.4.1. Disinfection practices for medical environments

**Fig. 11:**
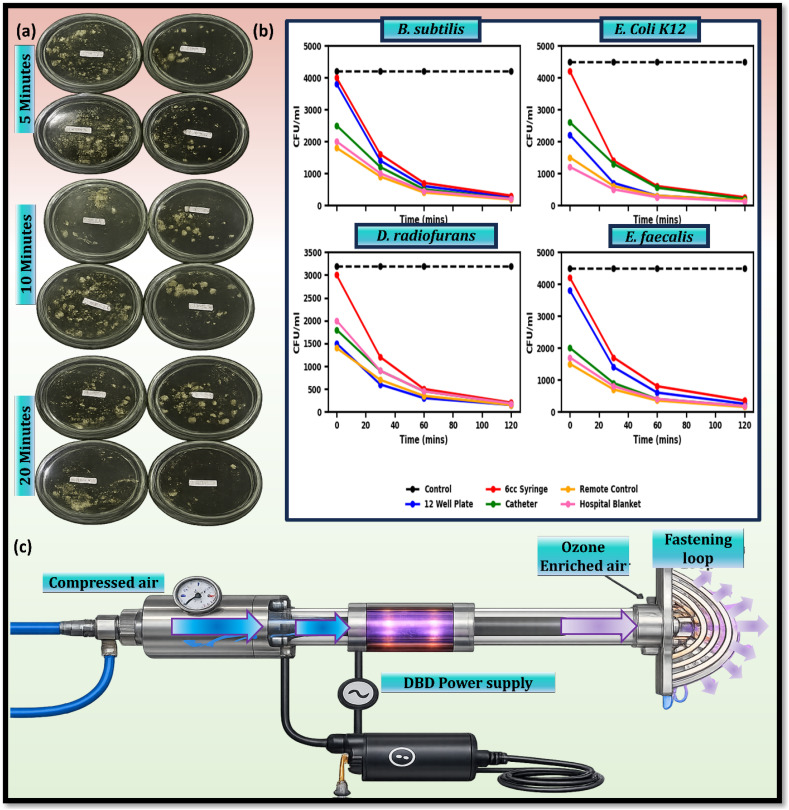
(a) Representative bacterial colonies grown on PCA medium following ozone exposure for 5, 10, and 20 min reproduced from *International Journal of Technology*.^206^ Copyright © 2017 Faculty of Engineering. (b) Ozone inactivation (kill) curves demonstrating a time-dependent reduction in bacterial load with increasing treatment duration. Reproduced from MDPI,^207^ © 2022, under a Creative Commons license. (c) Schematic illustration of the dielectric barrier discharge (DBD) reactor employed for face-mask sterilization experiments.^208^ Adapted from Springer Nature, 2021.^208^ © 2021 Springer Nature. Used under a Creative Commons license.

The use of ozone is also being considered as a viable method of disinfecting medical equipment and hospital setups. There is an increasing amount of literature that ozone when utilized under controlled conditions can be useful in the neutralisation of a wide range of bacterial and viral pathogens (Table 9). An example is the portable ozone sterilization device developed to target some of the most frequently used medical equipment, including a stethoscope and thermometer. Their experiment showed that the level of ozone of 4.94 ppm was enough to significantly decrease *Staphylococcus aureus* in a 20 minutes' exposure (Fig. 11a). On the same note, to investigated the efficiency of the use of ozone disinfection systems in the hospital equipment and the surrounding environments contaminated with organisms such as *Escherichia coli*, *Enterococcus faecal* is, *Bacillus subtilis*, radiation-resistant bacteria, and synthetic SARS-CoV-2 RNA. As represented in Fig. 11b, the microbial reduction was fast with the number of viable bacteria in the sample reducing to approximately 99 per cent after 2 hours, whereas the degradation of viral RNA was realized within 30–60 minutes.

**Table 9:** Molecular mechanisms of ozone-induced inactivation of pathogens

S. no.	Category	Year	Ozone form/method	Application object	Key outcomes	Ref.
1	Medical device sterilization	∼2000	Gaseous ozone (corona discharge)	Surgical instruments	Low-temperature, residue-free sterilization	198
2	Hospital surface disinfection	∼2005	Gaseous ozone	OT, ICU surfaces	Reduced microbial contamination	199
3	Healthcare water disinfection	∼2010	Aqueous ozone	Dialysis & hospital water	Rapid pathogen inactivation	200
4	Air disinfection	∼2015	Gaseous ozone	Hospital rooms	Lower airborne microorganisms	201
5	Dental disinfection	∼2015	Aqueous ozone	Dental tools	Biofilm removal, material safe	202
6	Wound protection	∼2020	Ozonated water/oils	Chronic wounds	Antimicrobial, faster healing	203
7	PPE disinfection	2020–21	Gaseous ozone (chambers)	Masks & gowns	Virus inactivation, reuse	204
8	Lab & medical waste treatment	2022–25	Gaseous/aqueous ozone	Lab tools, waste	Safe, eco-friendly disinfection	205

Ozone sterilization is gaining more and more interest as a way of disinfecting personal protective equipment and medical materials due to its ability to rapidly inactivate microorganisms without leaving most substrates visibly damaged. To studied a plasma-assisted ozone system that used a flow-through arrangement (Fig. 11c) and discovered that enhanced ozone penetration into mask fibres that enhanced disinfection performance significantly. The system showed a sterilization efficiency that was over 400 percent greater than that of the traditional sealed-chamber ozone treatments. To shown in another study that the dry ozone of 25 ppm concentration could completely inactivate the coronavirus surrogate HCoV-OC43 on N95 respirators and on the glass surfaces in 15 minutes. Medical polymers have also been reported to be compatible with ozone treatment. Indicatively, the indicated that ozone treatment was already shown to be effective in sterilizing polyhydroxybutyrate fibres without affecting its vital physicochemical characteristics. Comprehensively, the findings indicate that ozone is a quick and cheap alternative to disinfecting protective equipment with a minimum effect on material performance. However, impact of repeated ozone exposure on the filtration efficiency, mechanical stability and long run aging of the material are not well known. As these results are presumably dependent on the material composition and the solution design, more systematic investigations are needed to create the proper treatment conditions, and assess the durability during the different disinfection cycles.^209^

Ultrasonic treatment integrated into a combination of ozone-assisted sterilization was suggested as a low temperature variant of disinfecting medical equipment with a complex structure or low heat resistance. According to the invention of a system of dielectric barrier discharge ozone coupled with a 100 W ultrasonic bath, which was effective in microbial inactivation using a dissolved ozone concentration of 10 mg L^−1^ at room temperature (10–15 °C). In such arrangement the ultrasonic cavitation aids in physical removals of contaminants whereas ozone offers strong oxidative disinfection as well as enhances the work of sterilization without subjecting equipment to thermal stress. Though these are encouraging findings, these combined systems remain at experimental levels and very little information is available on the possibilities of scale-up, risks of occupational exposure, stability of operations and common clinical application.^210^

Generally, the existing research indicates that ozone can be used to inactivate a wide spectrum of bacteria and viruses on medical equipment, personal protective gear, and surfaces of the environment, especially in low-temperature and in locations that are hard to reach with conventional means. The key benefits of ozone disinfection are that it acts quickly as an antimicrobial agent, no lingering residues of the chemicals, and it is flexible to support different device geometries. But the majority of the results are based on controlled laboratory studies which underline the necessity of further confirmation in actual clinical situations. Subsequent studies should hence focus on the establishment of standardized levels of ozone, treatment timelines, system designs and safety levels so that they can permit uniform, dependable and clinically relocatable sterilization strategies.

#### 4.4.2. Therapeutic applications in clinical practice

Medical use of ozone has been growing in the recent years, as more and more evidence was provided to prove the effectiveness of ozone in managing microbial infections, inflammation, and tissue healing and regeneration. Table 10 provides a summary of the major clinical applications of ozone therapy. The use of ozone treatment has been investigated in various medical disciplines, most typically in the form of an adjunct or supplementary therapy as opposed to a main one. Ozone has shown antimicrobial and anti-inflammatory properties in the field of dentistry and advantages have been reported in the treatment of caries, periodontal, oral mucosal diseases, and in the treatment of selected maxillofacial diseases. Although cariogenic and periodontal pathogen reductions have been reported, the overall clinical outcome is similar or worse than more traditional antiseptics, and findings have been mostly based on small trials with a short-term follow-up.^211^

**Table 10:** Fundamental clinical basis of ozone therapy

S. no.	Disease area	Year (reported clinical use)	Ozone therapy type	Target condition	Main therapeutic effects	Ref.
1	Infectious diseases	1980s–present	Ozonated saline infusion	Sepsis, bacterial infections	Broad-spectrum antimicrobial action, immune modulation	94
2	Infectious diseases	1990s–present	Major autohemotherapy (MAH)	Viral infections (HBV, HCV, HIV adjunct)	Viral load reduction, improved antioxidant defence	103
3	Wound care & ulcers	1990s–present	Topical gaseous ozone	Diabetic foot ulcers	Enhanced wound healing, reduced microbial burden	109
4	Wound care & ulcers	2000s–present	Ozonated oils	Pressure sores, chronic wounds	Anti-inflammatory effect, tissue regeneration	129
5	Musculoskeletal disorders	1995–Present	Intramuscular ozone injection	Low back pain, disc herniation	Pain relief, reduced inflammation, muscle relaxation	138
6	Musculoskeletal disorders	2000s–present	Intra-articular ozone injection	Knee osteoarthritis	Improved joint mobility, analgesic effect	86
7	Neurological disorders	2000s–present	Rectal ozone insufflation	Multiple sclerosis (adjunct)	Improved oxygen metabolism, immune regulation	54
8	Cardiovascular diseases	1990s–present	Major autohemotherapy (MAH)	Peripheral arterial disease	Improved blood rheology, enhanced tissue oxygenation	186
9	Cardiovascular diseases	2000s–present	Ozonated saline infusion	Ischemic heart disease (adjunct)	Reduced oxidative stress, improved circulation	176
10	Dermatological diseases	1990s–present	Topical ozone therapy	Psoriasis, eczema	Anti-inflammatory, antimicrobial effects	34
11	Dental & oral health	2000s–present	Ozone gas/ozonized water	Dental caries, periodontitis	Bacterial eradication, enamel demineralization support	65
12	Gynaecological infections	2000s–present	Vaginal ozone insufflation	Vaginitis, candidiasis	Antifungal, antibacterial activity	55
13	Gastrointestinal disorders	2000s–present	Rectal ozone insufflation	Ulcerative colitis (adjunct)	Reduced inflammation, mucosal healing	183
14	Oncology (supportive care)	2000s–present	Major autohemotherapy (MAH)	Cancer-related fatigue	Improved oxygen delivery, immune stimulation	199
15	Metabolic disorders	2010s–present	Rectal ozone insufflation	Type 2 diabetes complications	Improved glucose metabolism, antioxidant balance	205

Ozone injections have demonstrated analgesic and functional effects in musculoskeletal and orthopaedic musculoskeletal diseases such as knee osteoarthritis and spinal diseases, and have comparable effects with conventional therapies. The fact that it is minimally invasive and has a good safety profile contributes to its use as a low cost supportive therapy, but methodological limitations and placebo effects are still issues. Chronic wound care has also been explored with experimental and clinical evidence supporting the use of ozone in improving tissue regeneration and microcirculation as well as in combination with conventional treatments. Ozone therapy has been shown to have immunomodulatory and supportive effects in neuroimmune diseases and infectious diseases, such as COVID-19, although the evidence is still preliminary because of heterogeneity, small sample sizes, and non-randomized design. Urology use has also been reported.^212^

All in all, the contemporary level of evidence regarding ozone therapy is of moderate to low quality, regardless of the positive mechanistic statistics and the clinical improvements that were reported. Before conclusive clinical recommendations can be made, standardized protocols, large randomized controlled trials and long term outcome data will be necessary.

Overall, the practical performance of ozone technologies in environmental and industrial applications depends on a balance between ozone generation efficiency, system integration, and operational safety. Comparative evaluation of generation methods indicates that plasma-based technologies, particularly dielectric barrier discharge systems, currently dominate large-scale industrial applications because of their high ozone yield and operational reliability.^134^ Electrochemical systems, although still developing, show strong potential for applications requiring compact design and high-purity dissolved ozone.^125,127^ Continued advances in electrode materials, reactor engineering, and intelligent monitoring systems are expected to improve energy efficiency and operational stability, thereby supporting the broader adoption of ozone-based technologies in sustainable environmental treatment processes.

## 5. Safety management strategies and regulatory controls

### 5.1. Process hazard analysis and risk management

The industrial ozone generation equipment has a variety of inherent safety hazards, which manifest themselves in the form of dependence on high electric power, reactive oxidants, and other supplementary chemical reactions. Among the key risks, high-voltage electricity exposure, ozone inhalation, hydrogen accumulation, and ignition, mercury release of UV lamps, and construction materials accelerated degradation can be mentioned. These risks are aggravated in applications of ozone where it is provided in the gaseous state, varying ozone concentrations, uneven distribution in occupied areas, and no control over human exposure. Therefore, a holistic process safety approach that has a systematic combination of hazard identification, risk analysis, preventive engineering control, and emergency preparedness is required in safe and compliant operation of all the ozone generation technologies.^213^

Process safety management is based on the identification of hazards. The use of structured analytical techniques, especially hazard and operability (HAZOP) analysis is often used to identify the discrepancies between the proposed operating condition and to determine their possible safety outcomes. Within the use of ultraviolet based ozone generators, the most possible hazards are exposures to UV radiation as well as mercury contamination due to lamp breaks, loss of system performance due to lamp aging and leaks. Some of the common deviations during operation include lamp failure, cooling failure and leakage of the irradiation chamber. Linear hazards that are related to the use of dielectric barrier discharge (DBD) ozone generators are primarily linked to electric shock due to high voltage components, unwanted arc creation, release of ozone, and deterioration or breakdown of dielectric materials. The risks usually relate to the deviations, namely, electrode relocation, inadequate thermal regulation, and unstable conditions of power supply. Electrolytic ozone generators present other safety issues such as hydrogen accumulation and the risk of explosion, membrane wear, sensitivity to purity of feed water, and overpressure. Typical defects with such systems are membrane failure, short circuiting, and the inappropriate electrolytes composition.^214^

Having identified hazards, it is usually evaluated through a probability-consequence framework where the overall risk is represented by the product of the probability and severity of the event (*R* = *P* × *C*). Table 11 indicates that rupture in UV ozone system of mercury lamps is typically considered as a low-frequency but high-impact, and thus the risk is classified as moderate. Electrical arcing and dielectric failure are regarded as a high-risk event in DBD generators and ozone leakage is more often referred to a moderate risk.^215^ In the case of electrolytic ozone systems, the most dangerous high-risk situations are hurricane and ignition of hydrogen, and the burst of membranes is usually considered to be a moderate risk. It is possible to identify the prevailing safety challenges and prioritize the focused mitigation strategies with the help of such risk stratification.

**Table 11:** Framework for safety risk assessment of ozone generation technologies

S. no.	Ozone generator	Major risk event	*P*	*C*	*R*	Risk level	Brief description
1	Corona discharge (CD)	Ozone gas leakage	4	5	20	Extreme	Seal or material failure releases high-concentration ozone, causing severe health hazards
2	Dielectric barrier discharge (DBD)	High-voltage exposure	3	4	12	High	High-voltage components pose electrical shock and fire risks during operation or maintenance
3	UV ozone generator	UV radiation exposure	2	4	8	Medium	Direct exposure to UV lamps may damage eyes and skin during servicing
4	Electrolytic ozone generator	Hydrogen gas accumulation	3	5	15	High	Hydrogen by-product may form explosive mixtures if ventilation fails
5	Plasma ozone generator	Plasma instability	3	4	12	High	Instability can cause sudden ozone spikes and electrical faults
6	Portable ozone generator	Indoor ozone overexposure	4	4	16	Extreme	Uncontrolled use easily exceeds safe indoor ozone limits

Engineering controls and safety-instrumented functions are major ways of risk reduction. Physical shielding against radiation, interlocked access panels, alarm and automatic shutdown are the means of protection in the UV-based systems in case of lamp or chamber failure. DBD ozone generators use high-voltage insulation, arc detectors, continuous ozone measurements, ventilation interlocks, redundant cooling units, as well as regular inspection and replacement of electrodes and dielectric parts.^216^ Electrolytic ozone systems have inbuilt hydrogen sensors, forced ventilation, fire suppressing systems, pressure relief, online water quality monitoring, and automatic shutdown logic to avoid dangerous operating conditions. Recent studies point to the fact that poor observation of the ozone concentration and environmental status raises considerably the probability of uncontrolled exposure, thus, raising the safety and regulatory risks. Emergency response planning is an urgent complement to preventive controls since it helps to mitigate residual risks that may not be entirely removed. In the case of UV ozone generator, emergency plans usually involve accidental UV exposure management, mercury spill management and clean-up, hazardous waste management and emergency shutdown of the generator. Examples of incidents in DBD installations include ozone leakage or electrical malfunction that are activated by alarms, ventilation systems and high-voltage isolation that are aided by electrical safety and respiratory protection personnel training. The electrolytic ozone systems are based on the continuous monitoring of hydrogen, forced ventilation, fire suppression, response in the event of spillage of electrolytes, as well as the automatic shutdown of power. It must continue to provide training to the operators, conduct regular inspections, and hold systematic maintenance audits to guarantee the long-term efficiency of such safety measures and reduce the risks to the workers, equipment, and the surrounding environment.^217,218^

### 5.2. Regulatory guidelines and enforcement

An effort to control the generation of ozone is a combination of international safety, environmental and industrial standards that govern the design, installation and operation of the ozone generation system. Occupational safety and machinery risk control is covered by such frameworks as ISO 45001 and ISO 12100, whereas electrical safety and appropriateness in high-risk industry environments are provided by ANSI/UL standards and European regulations, including ATEX and machinery directive.^219^ These requirements must be complied with to operate safely, gain legal approval, and acceptability in the global market. Recent research on gaseous ozone disinfection suggests that regulatory issues are not confined to equipment safety only. The main weakness is the lack of standard methods of defining conditions of ozone exposure. Poor reporting of concentration, contact time, humidity, and system design decreases comparability of studies and makes regulatory analysis difficult, hindering the implementation of gaseous ozone technology in disinfection and air treatment applications.

On installation of the system, regulatory approval usually comprises environmental impact assessment, approval of occupational safety and high voltage component inspection. Risk assessment, ventilation analysis, and confirmation of the ozone emissions and by-products to the regulatory limits are often part of the permit submissions.^220^ These are basically the measures that are concerned with safety rather than the variability of the performance of gaseous ozone systems in totality. Constant monitoring and documentation must be done during the operation in order to maintain compliance. The concentration of the ozone, electrical load, and hydrogen, as well as maintenance logs, incident and operator training records are to be documented.^221^ Still, the absence of standardized measurements of exposure, like standardized CT values is one of the biggest obstacles to regular regulatory evaluation of the treatment effectiveness. In general, the control of ozone incorporates safety requirements, authorization protocols, operational control, as well as equipment certification. In the case of gaseous ozone, the future regulatory acceptance will be based on the establishment of standardized definitions of exposure and performance evaluation to enable the construction of reliable and consistent implementation.

## 6. Current challenges and emerging trends

### 6.1. System integration of ozone technologies: technical and regulatory challenges

**Fig. 12:**
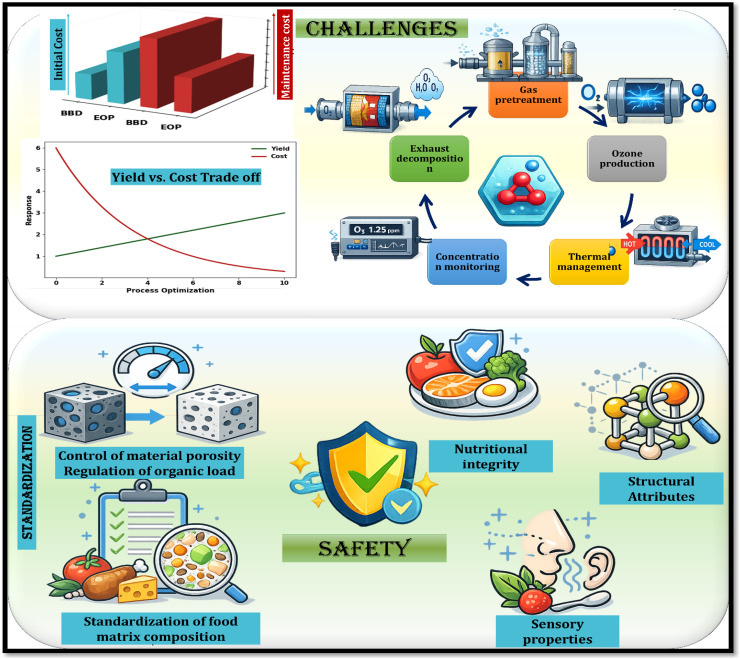
Key limitations and challenges of ozone technology in environmental applications.

Trade-offs between efficiency, cost and durability: although there have been long term research activities, ozone generation technologies continue to grapple with the chronic problems of simultaneously attaining high production efficiency, affordable operating costs and extended service life, as shown in Fig. 12a. Traditional systems of high-voltage discharge are highly prevalent because they require comparatively low cost of initial investment, but the discharge systems are often energy intensive, thus necessitating greater electricity consumption and consequent higher cost of operating with time.^222^ Besides, to reduce the creation of nitrogen oxides, such systems are frequently accompanied by complex gas pre-treatment mechanisms, which further complicate systems, load up their maintenance burden and total lifecycle cost. Another direction has been electrochemical generation of ozone which has the advantage of high purity and high concentration ozone without complex gas-handling procedures. However, its practical use in industry is still restricted by the high price and lack of durability of very important parts, especially high quality electrode materials and ion-conducting membranes. The cost of replacement of components is also high, as well as the material costs leading to high capital and maintenance costs. The development of the future should then be aimed at the joint performance of materials engineering and system architecture to increase the ozone production and reduce the cost and the lifespan of the operating time.^223^

System integration and adaptive operation control: as it is depicted in Fig. 12b, the stabilized functioning of modern ozone production systems is based on the successful integration of various sub-systems, such as gas conditioning, ozone synthesis, thermal control, real-time concentration monitoring, and exhaust gas treatment. These functional units have strong interactions, which complicate the precise control particularly when the operational conditions vary. Lack of coordination tends to result in fluctuating ozone levels, ineffective energy consumption and poor overall performance. Among the main problems is the unavailability of sensing devices that can sustain a long-term precision in an environment that is highly oxidative and humid. Simultaneously, several of the current control strategies are not very flexible and cannot effectively react to dynamic disturbances. Even though there are simple feedback regulation systems in place in some commercial systems, involving ozone sensors and generators, more sophisticated and intelligent control patterns are yet to be established. Advancement in sensors resistant to corrosion, adaptive control procedures and predictive maintenance technologies will play a critical role in enhancing stability in output, precision in dosing, energy conservation, and reliability of the system.^224^

Poor standardization and insufficient long-term safety experience: the further deployment of ozone-based technologies is also held back by the lack of application-specific assessment criteria that are standardized, which are as summarized in Fig. 12c. Several operational variables have a significant impact on ozone performance such as applied concentration, exposure time, humidity of the environment, and material properties (treated). Nevertheless, definitions and testing conditions that are used to present these parameters are frequently inconsistent across studies and are additionally problematic when comparing them directly, or assessing them through regulation. Besides this, despite the extensive knowledge on the efficacy of ozone in the inactivation of the microbes, extensive research to determine its long-term inactivation impacts on treated materials is still scarce. The possible effects on nutritional qualities, structural stability and sensory quality are often analyzed independently and without systematic assessment systems. The absence of standardized performance indicators and long-term safety data reduces the confidence of industries and slows down regulatory acceptance. It is thus necessary to have harmonized measurements of exposure, reproducible testing methods and application focused validation strategies to achieve fair and safe implementation of ozone technologies in industry.^225^

### 6.2. Future directions in convergent and intelligent systems

#### 6.2.1. Improvement in generation of gaseous ozone and development of hybrid systems

The current research directions should be focused on the gaseous ozone generation technologies as this research field still has some unanswered scientific questions and engineering constraints. Compared to systems using liquid-phase ozone, gas-phase operations have had very little development in integrated or hybrid systems. Specifically, the dielectric barrier discharge in combination with complementary methods like ultraviolet irradiation or plasma-assisted processes or catalytic enhancement is not investigated sufficiently. These combined strategies have obvious potential to improve oxidation activities, active reactive oxygen species generation, and restrict unneeded ozone disintegration. Further developments in dielectric materials, discharge architecture, and non-precious metal catalysts that do not pose an overall economic threat will be necessary to enhance the efficiency of the operation, long time stability and feasibility of the industry.

❖ Smart surveillance, operational algorithms, and the introduction of digital twins. Incremental developments are occurring with gaseous ozone systems moving away to smarter and adaptive operation as opposed to traditional automation. Inequality in the distribution of ozone and the unstable nature of the environment are other factors that lead to performance instability and can hardly be controlled by a static control mechanism. The future platforms must include the use of long life ozone sensing devices, real time monitoring of the environment and an adaptable feedback system to effectively manage the ozone concentration and time of exposure. Moreover, digital twin models with machine learning can assist predicting maintenance, optimizing operations, and improving safety supervision along life cycles within systems.

❖ Specific application and sustainability-based development of gaseous ozone technologies. The practice environment of gaseous ozone is projected to shift to more specific and contextual solutions. System designs that are flexible and modular can be customized to address individual requirements of food preservation, medical sterilization, air purification as well as industrial emission treatment. Simultaneously, the aspect of sustainability will be more significant and focus will be on energy-efficient operation, recyclable construction material and modular replacement of equipment. Standardized evaluation techniques will prove essential to the correlation of ozone exposure parameters and the outcomes of the treatment and enable the reliable implementation to industries.

## 7. Conclusions

The use of ozone technology is fast becoming a useful tool due to its strength of oxidation, environmental friendliness, and wide scope of utilization. As the world has shifted towards greener technologies and more efficient disinfection procedures, studies in this area have slowly shifted the focus out of individual performance improvement to the overall design of the system, precise process management, and standard practice. A summary of the essential characteristics of ozone, its production routes, and methods of its analysis suggests that efficiency and utility in the daily engineering practice are unimaginable without effective real-time monitoring. Even though ultraviolet irradiation, dielectric barrier discharge and electrochemical generation methods are all currently under intense research, each of these methods has some form of compromise in the aspects of energy efficiency, equipment life and the cost. Currently, there is no technology that can be used to provide at once the efficiency, low cost, and long term stability of operations. Consequently, the future development will probably depend on the hybrid generation approaches and the creation of modern functional materials. Ozone has been found to have great potential in water treatment, air purification, food processing and in the medical field in application oriented studies. Nevertheless, its performance is extremely reliant on the conditions of operation. Parameters like ozone concentration, exposure time, environmental traits, and interactions of the materials are strongly interconnected, which in most cases causes random results of the experiments and reduces its reproducibility with a scale increase. Specifically, in the case of gaseous ozone usage, the lack of unified performance metrics and standardized testing protocols is a key barrier in getting the regulation passed and the industry to embrace the technology. In addition to performance issues, there are safety concerns as well in the engineering implementation such as the high-voltage operation, ozone leakage, hydrogen build-up, and material aging. Current safety laws mainly relate to the operational practice and equipment design, and the systematic review of the long-term operation and safety risk has not been well examined. Consequently, the further evolution of the technology of ozone needs not only the improvements of the efficiency of the generation but also the significant enhancement of the framework of the risk assessment, monitoring, and regulatory norms. In the end, the real competition of the ozone-based systems will be based on their general reliability, safety, and consistency in the standard operating environment, but not the effectiveness of the generators. In this review, the problem-oriented approach is assigned and presents the major technical directions in the context of which the application of ozone technology is scalable, sustainable, and compliant.

## Conflicts of interest

The authors declare that there are no conflicts of interest.

## Funding

This work was supported and funded by the Deanship of Scientific Research at Imam Mohammad Ibn Saud Islamic University (IMSIU) (grant number IMSIU-DDRSP2603).

## Data Availability

No primary research results, software or code have been included and no new data were generated or analysed as part of this review.
